# Differential immunomodulatory effects of epirubicin/cyclophosphamide and docetaxel in breast cancer patients

**DOI:** 10.1186/s13046-023-02876-x

**Published:** 2023-11-14

**Authors:** Kerstin Wimmer, Monika Sachet, Cristiano Ramos, Sophie Frantal, Hanna Birnleitner, Christine Brostjan, Ruth Exner, Martin Filipits, Zsuzsanna Bago-Horvath, Margaretha Rudas, Rupert Bartsch, Michael Gnant, Christian F. Singer, Marija Balic, Daniel Egle, Rudolf Oehler, Florian Fitzal

**Affiliations:** 1 Department of General Surgery, Division of Visceral Surgery and Comprehensive Cancer Center, Medical University of Vienna, Waehringer Guertel 18-20, A-1090 Vienna, Austria; 2 Austrian Breast & Colorectal Cancer Study Group (ABCSG), Vienna, Austria; 3 Department of General Surgery, Division of Vascular Surgery, Medical University of Vienna, 1090 Vienna, Austria; 4 Center for Cancer Research, Medical University of Vienna, 1090 Vienna, Austria; 5 Department of Pathology, Medical University of Vienna, 1090 Vienna, Austria; 6 Comprehensive Cancer Center, Medical University of Vienna, Spitalgasse 23, 1090 Vienna, Austria; 7 Department of Medicine 1, Division of Oncology, Medical University of Vienna, 1090 Vienna, Austria; 8 Department of Gynecology, Medical University of Vienna, 1090 Vienna, Austria; 9 Department of Oncology, Medical University of Graz, Graz, Austria; 10 Department of Gynecology, Medical University Innsbruck, Innsbruck, Austria

**Keywords:** Neoadjuvant chemotherapy, Breast cancer, Immune cells, Epirubicin, Docetaxel, Immunomodulatory markers, Prediction of response to chemotherapy

## Abstract

**Background:**

Epirubicin/cyclophosphamide (EC) and docetaxel (D) are commonly used in a sequential regimen in the neoadjuvant treatment of early, high-risk or locally advanced breast cancer (BC). Novel approaches to increase the response rate combine this treatment with immunotherapies such as PD-1 inhibition. However, the expected stimulatory effect on lymphocytes may depend on the chemotherapy backbone. Therefore, we separately compared the immunomodulatory effects of EC and D in the setting of a randomized clinical trial.

**Methods:**

Tumor and blood samples of 154 patients from the ABCSG-34 trial were available (76 patients received four cycles of EC followed by four cycles of D; 78 patients get the reverse treatment sequence). Tumor-infiltrating lymphocytes, circulating lymphocytes and 14 soluble immune mediators were determined at baseline and at drug change. Furthermore, six BC cell lines were treated with E, C or D and co-cultured with immune cells.

**Results:**

Initial treatment with four cycles of EC reduced circulating B and T cells by 94% and 45%, respectively. In contrast, no comparable effects on lymphocytes were observed in patients treated with initial four cycles of D. Most immune mediators decreased under EC whereas D-treatment resulted in elevated levels of CXCL10, urokinase-type plasminogen activator (uPA) and its soluble receptor (suPAR). Accordingly, only the exposure of BC cell lines to D induced similar increases as compared to E. While treatment of BC cells with E was associated with cell shrinkage and apoptosis, D induced cell swelling and accumulation of cells in G2 phase.

**Conclusion:**

The deleterious effect of EC on lymphocytes indicates strong immunosuppressive properties of this combination therapy. D, in contrast, has no effect on lymphocytes, but triggers the secretion of stimulatory proteins in vivo and in vitro, indicating a supportive effect on the immune system. Underlying differences in the induced cell death might be causal. These divergent immunomodulatory effects of epirubicin/cyclophosphamide and docetaxel should be considered when planning future combinations with immunotherapies in breast cancer.

**Supplementary Information:**

The online version contains supplementary material available at 10.1186/s13046-023-02876-x.

## Background

Despite ongoing improvements in screening programs and increasing awareness, breast cancer (BC) remains a significant cause of death [1]. Thus, research on therapeutic options as well as further development of personalized treatment approaches is urgently needed. Neoadjuvant chemotherapy (NAC) with a sequential administration of anthracyclines and taxanes is currently one standard-of-care therapy option in patients with early, high-risk or locally advanced hormone receptor positive or triple negative BC. NAC is nowadays widely applied, but not all patients profit from this treatment. Continuous rise of interest in the immune system as a potent anti-tumor defense was observed. A strong infiltration of the tumor with immune cells correlates with a better prognosis in certain cancer types [2–5]. Especially cytotoxic CD8^+^ T cells and NK cells have been associated with beneficial anti-cancer immune response. In BC, a higher number of stromal CD8^+^ lymphocytes were associated with improved survival [4]. Increasing tumor-infiltrating lymphocytes (TILs) during taxane-based NAC correlated with favorable tumor response [6]. TNBC is known to provoke the most abundant immune cell infiltration in breast cancer. This underlines the utmost importance of cytotoxic immune cells in BC control. The balance between anti- and pro-inflammatory molecules in the tumor microenvironment and in the circulation and their interaction with immune cells determine the activity level of T cells. On the one hand, the tumor microenvironment can shift T cells into an anergic state whereas on the other hand, pro-inflammatory mechanisms—provoked by chemotherapy-induced immunogenic cell death or by other immunomodulatory factors – can stimulate them [7]. Immunogenic cell death activates the immune system and also promotes a tumor-specific adaptive immune response [8]. Several studies showed that chemotherapy-induced cell death was associated with increased tumor immune cell infiltration [9].

Immunotherapies such as immune checkpoint inhibitors, adoptive T-cell immunotherapy, and tumor vaccine immunotherapy became promising novel treatment approaches in TNBC [10]. Complementing traditional chemotherapy with immunotherapy may exert synergistic effects. The KEYNOTE-522 trial confirmed that the addition of pembrolizumab, a PD-1 inhibitor, to anthracycline and taxane chemotherapy can increase pathologic complete response (pCR) rates in early stage TNBC [11] while the combination of carboplatin and taxane based chemotherapy with atezolizumab in the NeoTRIP Michelangelo trial showed no advantages [12]. Similarly, the combination of chemotherapy with a PD-L1 inhibitor in the phase II GeparNuevo trial was also negative for the primary endpoint pCR, but suggested improved invasive DFS, distant DFS, and OS in PD-L1 immune cell-positive BC [13, 14].

Activity of immunotherapy might be influenced by the chemotherapy backbone and particular attention must be paid to the complex interplay of chemotherapy and the immune system. The question arises, if chemotherapeutic agents differ in their influence on immune cells and hence, if the administered chemotherapeutic agents are equally compatible with immune-related therapies. For the here presented analysis, aiming to determine the particular effects of either epirubicin/cyclophosphamide (EC) or docetaxel (D) on immune cells without the interference of previous treatments, the ABCSG-34 study served as a basis. In this trial patients with BC of luminal or triple negative subtype were included and received either upfront EC followed by D or a reverse sequence with upfront D and then EC [15]. Although no differences in terms of pCR rates or residual cancer burden (RCB) score were observed between the two sequencing groups [16], the study design and a comprehensive pre-planned translational program render the ABCSG 34 cohort an ideal platform to study cytotoxic and immune-mediated therapy effects. The present analysis focused on the effects of EC and D on lymphocytes in the tumor microenvironment as well as in the circulation and on soluble immunomodulatory molecules. Therefore, we analyzed the initial cycles of NAC before the switch of drugs. This unique design allowed for a direct comparison of the distinct chemotherapeutic agents in the setting of a randomized clinical trial.

## Methods

### Patient populations, study design and sample collection

**Fig. 1:**
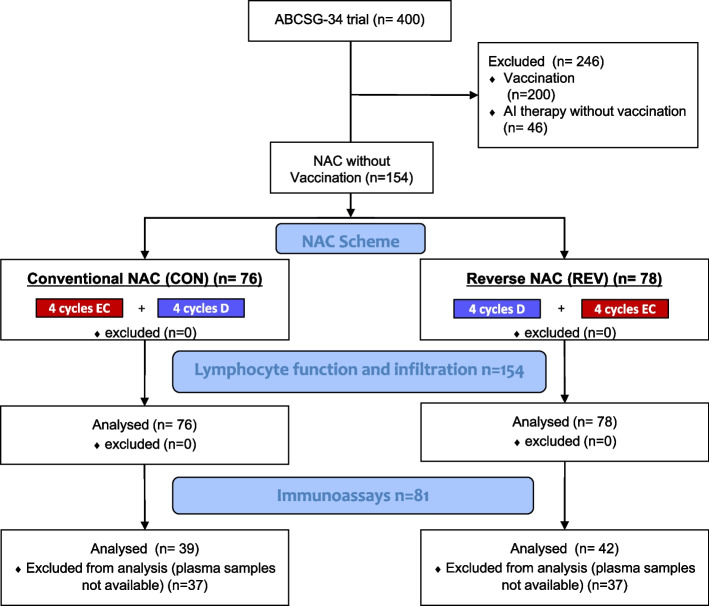
Patient selection diagram. This study was based on the ABCSG-34 trial which included a total of 400 patients. Half of them got a vaccination, which was out of the scope of the current study, and they were therefore excluded. Additionally, we excluded non-vaccinated patients treated with aromatase inhibitors (AI). The remaining 154 patients were randomly assigned to two treatment arms with different sequences of neoadjuvant chemotherapy (NAC): the conventional sequence (CON, *n* = 76) with 4 cycles of EC followed by 4 cycles of D and the reverse sequence (REV, *n* = 78) with first D then EC. From this collective (*n* = 154) we determined lymphocyte function (i.e. PHA-induced IFNγ production of circulating lymphocytes) and infiltration (i.e. TILs). Immunoassays for characterization of lymphocyte subpopulation (e.g. CD3 and CD19) and for quantification of soluble immune mediators were started later and were therefore only determined in a subgroup (*n* = 81)

The already completed ABCSG-34 trial, conducted by the Austrian Breast Cancer and Colorectal Surgery Group (ABCSG), served as basis [15]. The trial addressed pre- and postmenopausal female patients with early primary invasive BC without HER2-overexpression. It was a prospectively, randomized, open, 2-arm, multicentre, phase-II study in 400 breast cancer patients, treated with or without a therapeutic cancer vaccine (L-BLP25, Stimuvax®) in the preoperative setting. Patients were 1:1 randomized to preoperative standard of care (SoC) treatment with or without vaccine-BLP25. The preoperative SoC consisted of chemotherapy or endocrine therapy with an aromatase inhibitor (AI). AI therapy was considered as SoC in postmenopausal women with intermediate (+ +) or high (+ + +) estrogen receptor (ER) expression, a grading of 1, 2 or X, and a Ki67 of < 14%. Women with TNBC, premenopausal women, patients with absent (-) or low ER expression ( +) and patients with G3 tumors were treated with anthracycline and taxane-based NAC as SoC. The chemotherapy, consisting of EC and D, was either administered in a conventional (CON) or in a reverse (REV) sequence. In the conventional sequence, 4 cycles of EC were followed by 4 cycles of D in 3-week intervals whereas 4 cycles of D were followed by 4 cycles of EC in 3-week intervals in the reverse scheme. For the assessment of tumor response to preoperative therapy, the residual cancer burden (RCB) – prognostic for long-term survival in BC patients after NAC – was used [17]. RCB 0 (i.e. pathological complete response) was confirmed by central pathological review. The here presented study is a post hoc biomarker analysis conducted in the subset of patients accrued to the chemotherapy cohort and randomized to SoC. We created two investigational sets, the “lymphocyte function & infiltration” and the “immunoassay” subsets (see Fig. 1). Patients were selected for the “lymphocyte function & infiltration” subgroup if whole blood analysis as well as lymphocyte subtyping data was available at baseline, midterm and the time point operation. In this set we analyzed the influence of NAC on white blood cells, leukocyte subtypes, the IFNγ production and stromal (s) as well as invasive (i) TILs. In the “immunoassay” subset we investigated the effect of NAC on 14 different soluble parameters in plasma samples. For this subset, patients of the “lymphocyte function & infiltration” group were selected if plasma samples at all three time points (i.e. baseline, midterm, operation) were available. Research biopsies as well as blood samples were obtained before treatment (baseline or B), at midterm (M; i.e., after either 4 cycles of EC or 4 cycles of D), and after the second 4 cycles of therapy before surgery (S). Blood was collected in two different types of tube: (i) in EDTA containing tubes (Greiner Bio-one, Kremsmünster, Austria) for white blood cell (WBC) counting, leukocyte subtyping and plasma preparation (ii) in BD CPT™ tubes (BD, Heidelberg, Germany) for lymphocyte stimulation assays.

### Quantification of iTILs and sTILs

Tumor-infiltrating lymphocytes (TILs) were quantified according to the recommendations by the International TILs Working Group published in 2014 [18] with modifications according to Denkert et al. 2018 [19]. TILs were quantified on H&E sections of core biopsies obtained at the indicated time. Intratumoral and stromal TILs (iTILs and sTILs, respectively) were measured as percentage of mononuclear immune cells within or in between tumor cell nests, respectively. Three predefined categories were used: low TILs (0–10%), intermediate TILs (11–59%), or high TILs (60–100%). Examples are shown in Supplementary Figure S1.

### White blood cell counting and leukocyte subtyping

The total WBC count was determined in an aliquot of the EDTA blood using a Sysmex hematologic analyzer (Sysmex Austria, Vienna, Austria). For leukocyte subtyping red blood cells were lysed in another aliquot of the EDTA blood, and the remaining leukocytes were stained with fluorescently labelled antibodies against CD3, CD4, CD8, CD14, CD15, and CD19 (all from Thermo Fisher Scientific, Vienna, Austria). Sample acquisition was performed on a Gallios Flow Cytometer (Beckman Coulter, Indianapolis, IN, USA) and data was analyzed using the Kaluza 2.1 software (Beckman Coulter). See Supplementary Figure S2 for the gating strategy.

### T cell stimulation

Peripheral blood mononuclear cells (PBMCs) were prepared from the BD CPT™ tubes (BD, Heidelberg, Germany) according to the instructions by the manufacturer. Then phytohemagglutinin (PHA) was added for T cell stimulation and the tubes were incubated at 37 °C for 18 h. Stimulated T cells were defined as the percentage of IFNγ producing CD8^+^ T cells. Therefore, PBMCs were first stained with antibodies against CD3 and CD8, then permeabilized and stained for intracellular IFNγ. All antibodies were from Thermo Fisher Scientific (Vienna, Austria). The percentage of CD3^+^/CD8^+^/IFNγ^+^ cells was determined by flow cytometry using a Gallios Flow Cytometer (Beckman Coulter, Indianapolis, IN, USA) and data was analyzed using the Kaluza 2.1 software (Beckman Coulter). See Supplementary Figure S2 for gating strategy.

### Soluble immunomodulatory factors

Plasma was prepared by centrifugation of the remaining EDTA blood and stored in aliquots at -80 °C. In a pilot experiment, EDTA plasma of eight non-vaccinated patients before treatment initiation with AI was investigated. Therefore, an Olink® assay was performed which used matched pairs of oligonucleotide-labelled antibodies that were subsequently quantified by standard real-time PCR (Olink AB, Uppsala, Sweden). This enabled the simultaneous analysis of 92 different immunomodulatory parameters (for a detailed list of all factors see Supplementary Figure S3). The assay was performed according to the instructions of the manufacturer. Referring to the 30 most abundant molecules determined in the Olink® pilot assay, we selected 14 immunomodulatory parameters according to their oncological relevance and their association with BC as revealed by the literature. The resulting list included urokinase (uPA) and its soluble receptor (suPAR), vascular endothelial growth factor (VEGF-A), osteoprotegerin (OPG), monocyte chemoattractant protein 1 (MCP1 or CCL2), monocyte chemoattractant protein 2 (MCP2 or CCL8), macrophage colony-stimulating factor (M-CSF or CSF1), TNF-related weak inducer of apoptosis (TWEAK or TNFSF12), TNF-related apoptosis-inducing ligand (TRAIL or TNFSF10), interferon gamma-induced protein 10 (IP-10 or CXCL10), eotaxin-1 (or CCL11), T cell immunoglobulin and mucin-domain containing-3 (TIM-3), soluble CD27 (sCD27), and programmed cell death 1 ligand 2 (PD-L2). The majority of the selected immunomodulatory factors were analyzed by ProcartaPlex™ multiplex immunoassay (Thermo Fisher Scientific, Vienna, Austria). Plates were assessed using the Luminex 200 System (Luminex Corp. Austin, TX, USA). Two factors, uPA and suPAR, were analyzed by individual ELISA assays (Thermo Fisher Scientific, Vienna, Austria). The ELISA and the multiplex assays were performed in accordance with the manufacturer's instructions.

### Cancer cell line treatment and analysis

To represent the different BC subtypes, six cell lines were selected for in vitro experiments: MCF-7 and ZR-75–1 (both luminal A), BT-474 (luminal B), HCC-197 and HCC-1143 (both TNBC) and SK-BR-3 (HER2 +) [20, 21].The cell lines were obtained from ATCC (https://www.atcc.org), and were tested for mycoplasma contamination regularly, within two weeks before the cell culture models were established. MCF-7 and SK-BR-3 were grown in DMEM containing 10% fetal bovine serum (FCS) whereas all other cells were cultured in RPMI medium containing 10% FCS. The duration of the cell culture assay was five days. On day 1, cells were washed with phosphate-buffered saline (PBS, 1x, pH 7.4), counted and 40.000 cells were seeded in 500µL culture medium with 10% FCS in 24-well plates. On day 2, cells were washed with PBS, and chemotherapy was added into the appropriate wells at a final concentration of 2 µM for epirubicin, 15 µM for the cyclophosphamide metabolite 4OOH-CY, and 1 µM for docetaxel (diluted with the respective medium). Per cell line, two wells remained untreated. On day 4, 48 h after incubation with chemotherapy, pictures were taken and WBCs were isolated by red cell lysis. Per well, 500 000 WBCs were added. WBCs were added either onto a BC cell culture or into the supernatant of the respective cell culture. On day 5, the supernatants were transferred from wells into pre-cooled µFACS tubes. Per well, 2 × 120 µl aliquots were stored at -80 °C. Supernatants of untreated tumor cells, treated tumor cells, treated tumor cells in co-culture with WBCs and of WBCs alone were analyzed for soluble immunomodulatory factors by multiplex bead array immunoassay or ELISA (see above). The cell cycle of tumor cells was analyzed using a Propidium Iodide Flow Cytometry Kit according to the instructions by the manufacturer (Abcam, Cambridge, UK). Caspase 3 activation was determined using specific antibodies (559,565, BD Pharmingen, Franklin Lanes, NJ, USA) as described previously [22, 23].

### Statistics

All analyses were based on the intention-to-treat principle. Hence, patients were analyzed according to their randomized treatment. Patient characteristics are presented descriptively for patients with CON and REV and in total. “Lymphocyte function & infiltration” and “immunoassay” parameters at baseline, as well as changes in parameters between baseline and mid-therapy are presented and compared descriptively between patients with CON vs REV, RCB 0/I (≤ 1.36) vs. RCB II/III (> 1.36) and pCR yes vs. no by Wilcoxon tests. Scatterplots and boxplots show the change over time from baseline to surgery. A possible predictive role of “lymphocyte function & infiltration” parameters on RCB or pCR was assessed by logistic regression models. Primary analysis models included treatment arm only. Covariable models included treatment arm and the respective covariable (changes between baseline and mid-therapy) and were adjusted for the according covariable baseline value. Extended models additionally included a treatment-by-covariable interaction. Odds ratios (OR) with 95% confidence interval (CI) are provided. Measured parameters in the in vitro experiment of three BC cell lines in co-culture were presented descriptively. For each parameter, measurements were performed twice and the given value represents the mean. As the main ABCSG-34 study was planned for different objectives/endpoints, all results found in this subgroup analysis need to be considered exploratory. Analyses were carried out using the statistical analysis system (SAS) software (version 9.3 or higher) and GraphPad Prism (version 8.0.2.).

## Results

Of the 400 participating ABCSG-34 trial patients, 246 patients were excluded from the here presented analysis due to their randomization (see Fig. 1). The exclusion of 200 patients, who were vaccinated with LBLP-25, and 46, who were treated with AI-therapy, resulted in 154 patients that were selected for the “lymphocyte function & infiltration” subset. Of those, 81 patients were selected for further analyses in the “immunoassay” subgroup.

### Predictive value of tumor-infiltrating lymphocytes under NAC

In the “lymphocyte function & infiltration” subset, 76 patients were treated with NAC in a conventional sequence (CON) whereas 78 were treated reversely (REV, see Fig. 1). Further patients’ characteristics of this subset are shown in Table 1.

**Table 1:** Patient characteristics of the 154 patients selected from the ABCSG-34 trial

	*CON (n* = *76)*	*REV (n* = *78)*	*Total (n* = *154)*
Age (years)			
Median (min–max)	48 (26–78)	47 (26–75)	47 (26–78)
BMI			
Median (min–max)	25 (18–35)	25 (19–41)	25 (18–41)
Menopausal status – n (%)			
Perimenopausal	29 (38.2%)	28 (35.9%)	57 (37.0%)
Postmenopausal	3 (3.9%)	3 (3.8%)	6 (3.9%)
Premenopausal	44 (57.9%)	47 (60.3%)	91 (59.1%)
T-stage – n (%)			
T1	18 (23.7%)	24 (30.8%)	42 (27.3%)
T2/T3/T4	58 (76.3%)	54 (69.2%)	112 (72.7%)
Triple negative – n (%)			
No	41 (53.9%)	47 (60.3%)	88 (57.1%)
Yes	27 (35.5%)	27 (34.6%)	54 (35.1%)
Missing	8 (10.5%)	4 (5.1%)	12 (7.8%)
N-stage – n (%)			
Negative	41 (53.9%)	39 (50.0%)	80 (51.9%)
Positive	33 (43.4%)	39 (50.0%)	72 (46.8%)
Missing	2 (2.6%)	0 (0.0%)	2 (1.3%)
M-stage – n (%)			
M0	76 (100.0%)	78 (100.0%)	154 (100.0%)
Grading – n (%)			
G1	1 (1.3%)	1 (1.3%)	2 (1.3%)
G2/Gx	23 (30.3%)	23 (29.5%)	46 (29.9%)
G3	49 (64.5%)	52 (66.7%)	101 (65.6%)
Missing	3 (3.9%)	2 (2.6%)	5 (3.2%)
Her2 – n (%)			
Negative	64 (84.2%)	67 (85.9%)	131 (85.1%)
Positive	0 (0.0%)	1 (1.3%)	1 (0.6%)
Missing	12 (15.8%)	10 (12.8%)	22 (14.3%)
ER – n (%)			
Negative	37 (48.7%)	35 (44.9%)	72 (46.8%)
Positive	36 (47.4%)	42 (53.8%)	78 (50.6%)
Missing	3 (3.9%)	1 (1.3%)	4 (2.6%)
PgR – n (%)			
Negative	36 (47.4%)	35 (44.9%)	71 (46.1%)
Positive	37 (48.7%)	42 (53.8%)	79 (51.3%)
Missing	3 (3.9%)	1 (1.3%)	4 (2.6%)
Ki67			
N	73.0	74.0	147
Median (min–max)	50 (3–90)	50 (3–90)	50 (3–90)
Surgery Type – n (%)			
Breast conserving	46 (60.5%)	43 (55.1%)	89 (57.8%)
Mastectomy	21 (27.6%)	24 (30.8%)	45 (29.2%)
Other	6 (7.9%)	6 (7.7%)	12 (7.8%)
Skin sparing mastectomy	2 (2.6%)	3 (3.8%)	5 (3.2%)
Missing	1 (1.3%)	2 (2.6%)	3 (1.9%)
Sentinel node biopsy – n (%)			
No	29 (38.2%)	27 (34.6%)	56 (36.4%)
Yes	47 (61.8%)	49 (62.8%)	96 (62.3%)
Missing	0 (0.0%)	2 (2.6%)	2 (1.3%)
Axilla dissection – n (%)			
No	33 (43.4%)	29 (37.2%)	62 (40.3%)
Yes	43 (56.6%)	47 (60.3%)	90 (58.4%)
Missing	0 (0.0%)	2 (2.6%)	2 (1.3%)
RCB – n (%)			
RCB 0	16 (21.1%)	15 (19.2%)	31 (20.1%)
RCB I	10 (13.2%)	13 (16.7%)	23 (14.9%)
RCB II	34 (44.7%)	30 (38.5%)	64 (41.6%)
RCB III	10 (13.2%)	15 (19.2%)	25 (16.2%)
Missing	6 (7.9%)	5 (6.4%)	11 (7.1%)
pCR – n (%)			
No	58 (76.3%)	59 (75.6%)	117 (76.0%)
Yes	17 (22.4%)	16 (20.5%)	33 (21.4%)
Missing	1 (1.3%)	3 (3.8%)	4 (2.6%)

*N* number of patients in ITT analysis set, *CON* conventional neoadjuvant chemotherapy scheme, *REV* reverse neoadjuvant chemotherapy scheme, *SD* standard deviation, *Min* Minimum, *Max* Maximum, *BMI* body mass index, *Her2* human epidermal growth factor receptor 2, *ER* estrogen receptor, *PgR* progesterone receptor, *RCB* Residual Cancer Burden, *pCR* pathologic complete response. For patients who experience bilateral cancer information from the worse side is used for descriptive summaries

**Fig. 2:**
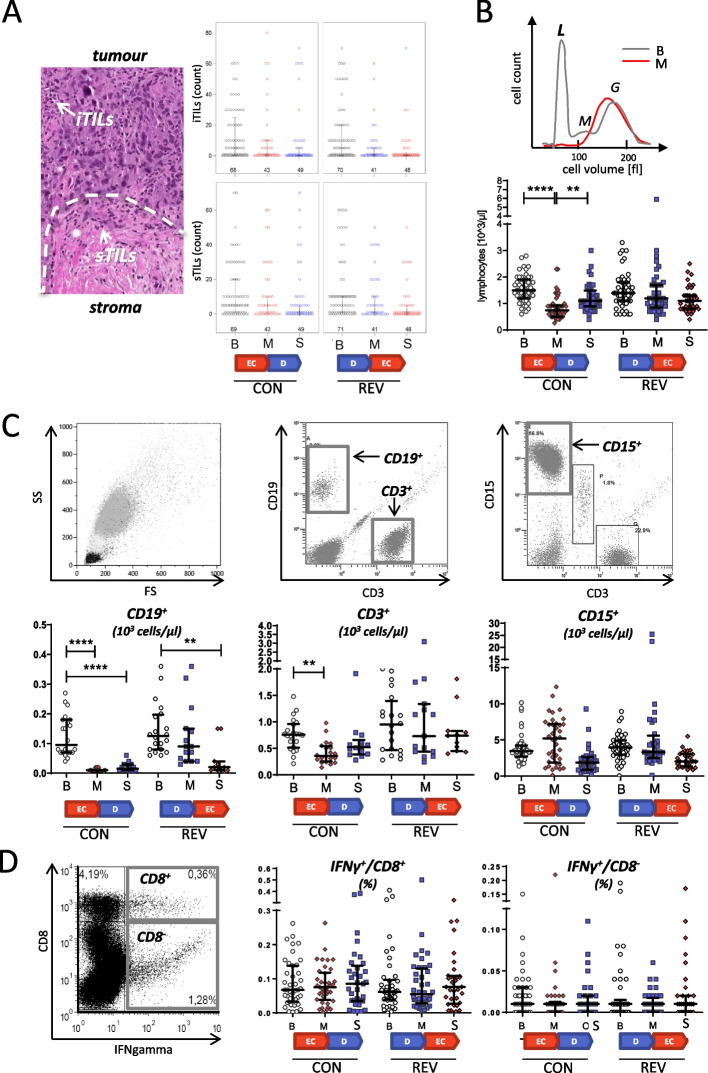
Effect of NAC on lymphocyte count and function. Tumor tissues and blood samples were collected at baseline (B), mid-therapy (M), and surgery (S) from patients treated with either conventional (CON) or reverse (REV) sequence of NAC. The color indicates the therapy administered immediately prior to the respective analysis: red for EC and blue for D. The pre-treatment analysis (baseline, B) is indicated in black. **A** Histological quantification of intratumoral TILs (iTILs) and stromal TILs (sTILs) in tumor tissue samples. The left panel shows an H&E stained tumor tissue. The invasive margin of the tumor is indicated by a dashed line. The arrows indicate examples of TILs. The panels on the right show the quantitative analysis. **B** Hemocytometric analysis of blood samples: The top graph shows typical examples of such analyses in blood samples taken at baseline (B) and midterm (M) of a CON patient. The lines indicate the cell volume distribution of the WBC populations (lymphocytes, L; monocytes, M; granulocytes, G). The bottom graph summarizes the lymphocyte counts of all patients. **C** Flow cytometric lymphocyte subtyping: quantification of CD3^+^ T cells, CD19^+^ B cells, and CD15^+^ granulocytes. **D** Lymphocyte function: PBMCs were prepared from peripheral blood and stimulated with PHA. IFNγ^+^/CD8^+^ T cells (left) and IFNγ^+^/CD8^−^ cells (right) were counted by flow cytometry. All graphs show individual patient values as well as median ± IQR. The statistical significance was calculated using a paired Student’s t-test (**… ≤ 0.01; ****… ≤ 0.001)

First, tumor tissue was analyzed for the purpose of comparing the effect of EC and D on TILs. Regarding the change of lymphocytes during the first four cycles of NAC, neither the initial four cycles of EC nor of D had effects on lymphocytes in the tumor microenvironment (see Fig. 2A and Supplementary Table S1). Furthermore, the response of the tumor to NAC measured by the RCB-score or the rate of pCR didn’t differ between CON and REV NAC scheme. The CON arm included 34.2% responders (RCB score of ≤ 1.36) and the REV arm 35.9%. A pCR could be detected in 21.4% of all patients and was equally distributed between CON and REV (22.4% and 20.4%, respectively). Thus, both treatment arms resulted in similar response rates.

Independently of the treatment sequence (CON or REV), responders and non-responders differed greatly in terms of TILs. During the initial 4 cycles of NAC an increase of sTILs and iTILs was observable in patients with an RCB ≤ 1.36 (*n* = 12; median =  + 17.5% and + 2.0%, respectively). Patients with a higher RCB (*n* = 62) showed a different trend (-1.0% *p* = 0.001 and -1.0% *p* = 0.023, respectively; see Supplementary Table S2). Patients with (*n* = 6) and without pCR (*n* = 69) showed a similar dynamic regarding TILs (sTILs: + 20% vs. 0.0% *p* = 0.002; iTILs 9.5% vs. -1.0% *p* = 0.088). These changes revealed that the lymphocyte infiltration of the tumor microenvironment could be modulated by four cycles of NAC between baseline and midterm.

As NAC-induced changes of sTILs and iTILs differed between responders and non-responders, we evaluated whether they predicted response to therapy applying two different logistic regression models. The first model shown in Table 2 included either the treatment effect alone or combined with NAC-induced changes between baseline and midterm in sTILs and iTILs, respectively, as covariables. It showed that higher changes increased the chance for RCB 0/I and pCR. The second model investigated the interactions between treatment and sTILs and iTILs, respectively. It revealed that only changes of sTILs and iTILs in the CON arm were considerable, indicating an exclusive effect of initial EC, whereas D showed no effect.

**Table 2:** Model 1: Logistic regression models including treatment arm and NAC-induced changes between baseline and midterm in sTILs and iTILs. a Based on logistic regression analysis including treatment arm; Odds ratio > 1 favours reverse chemotherapy. b Based on logistic regression analysis including treatment arm and the respective covariate and adjusted for the respective covariate baseline value. Odds ratio > 1 for the covariate favours increased differences (values at midtherapy are higher than values at baseline). Model 2: Logistic regression models including interactions between treatment arm and NAC-induced changes between baseline and midterm in sTILs and iTILs sTILs and iTILs. n = Number of subjects who observed an event; OR = Odds Ratio; B = baseline; M = midterm. a Based on logistic regression analysis including treatment arm, the respective covariate and treatment-by-covariate interaction and adjusted for the respective covariate baseline value

Model 1^a^										
	patients with RCB ≤ 1.36 (vs. RCB > 1.36)					patients with pCR (vs. no pCR)				
		Covariate Effect (Odds Ratio)		Treatment Effect (Odds Ratio)			Covariate Effect (Odds Ratio)		Treatment Effect (Odds Ratio)	
Covariates added to the primary analysis model^b^	n	Pt. Est. (95% CI)	p	Pt. Est. (95% CI)	p	n	Pt. Est. (95% CI)	p	Pt. Est. (95% CI)	p
	142			1.015 (0.514, 2.005)	0.965	150			0.925 (0.427, 2.004)	0.843
Change B to M in Stromal TILs	73	1.094 (1.036, 1.155)	0.001	1.146 (0.255, 5.158)	0.859	75	1.102 (1.032, 1.175)	0.004	0.677 (0.087, 5.298)	0.71
Change B to M in Intratumoral TILs	73	1.066 (1.018, 1.116)	0.006	1.073 (0.257, 4.482)	0.924	75	1.071 (1.018, 1.126)	0.008	0.567 (0.075, 4.320)	0.58
Model 2										
	patients with RCB ≤ 1.36 (vs. RCB > 1.36)					patients with pCR (vs. no pCR)				
Covariates and interactions added to the primary analysis model^a^		Covariate Effect (Odds Ratio)					Covariate Effect (Odds Ratio)			
	n	Pt. Est. (95% CI)			p	n	Pt. Est. (95% CI)			p
Change B to M in Stromal TILs	73					75				
CON	38	1.147 (1.036, 1.269)			0.0080	28	1.117 (1.024, 1.217)			0.0122
REV	35	1.044 (0.963, 1.131)			0.2985	37	1.069 (0.953, 1.200)			0.2542
Change B to M in Intratumoral TILs	73					75				
CON	38	1.059 (1.007, 1.112)			0.0245	38	1.061 (1.008, 1.117)			0.0242
REV	35	1.096 (0.985, 1.219)			0.0933	37	1.181 (0.978, 1.425)			0.0842

### Epirubicin-based chemotherapy suppresses lymphocytes whereas docetaxel maintains their number and function

The differential blood count showed a strong decrease of lymphocytes in patients treated with the initial four cycles of EC (from 1.57 × 10^3^ ± 0.52 cells/µl to 0.86 × 10^3^ ± 0.42 cells/µl; *p* < 0.001; see Fig. 2B). In contrast, initial D had no effect on the lymphocyte count. The great majority of lymphocytes are T cells, followed by B cells and much fewer natural killer cells. A detailed analysis by flow cytometry showed an EC-induced decrease of CD19^+^ B cells which persisted during the subsequent 4 cycles of D until completion of NAC (see Fig. 2C). A similar drop was also observed when EC was administered after D. The reduction of B cells did not differ between responders and non-responders (see Supplementary Figure S4 and Supplementary Table S3). Hence, EC seemed to have a sustainable and long-lasting suppressive effect on B cells. The decrease of CD3^+^ T cells when EC was administered initially was less pronounced compared to the one of B cells and was not observed when EC was given after D. In contrast, the initial 4 cycles of D in the REV arm did not reduce the number of circulating T or B cells. Remarkably, CD15^+^ granulocytes did not show statistically significant changes in their cell number in response to the different chemotherapy treatments. Similarly, we found no change in CD14^+^ monocytes or CD56^+^ NK-cells (data not shown). Because T cells are essential for an effective anti-tumor immune response, we investigated T cell function. Therefore, we isolated PBMCs from blood samples collected at baseline, midterm and surgery. They were then treated ex vivo with PHA and the ability of cytotoxic T cells to produce IFNγ was measured by flow cytometry. Neither EC nor D showed any effect on this T cell function (see Fig. 2D).

Lymphocytes may be regulated by a variety of different cytokines, chemokines and other immunomodulatory factors. Hence, we investigated such molecules in our patient collective. For this purpose, 81 patients of the “lymphocyte function & infiltration” subset were further selected for the “immunoassay” subgroup. Fourteen above mentioned factors were measured in blood samples at baseline, midterm and at surgery. Five factors (uPA, CXCL10, sTNFSF10, suPAR, and CCL8) reacted differently to EC as compared to D (see Table 3; a detailed illustration for every single factor is shown in Supplementary Figure S5 A + B). All of them decreased in response to four cycles of EC, which is in accordance with the suppressive effect of this treatment described above. Interestingly, uPA and its soluble receptor suPAR as well as CXCL10 increased in D-treated patients.

**Table 3:** Parameter changes between baseline and midterm in patients with conventional (CON) or reverse (REV) NAC. A stronger decrease of some parameters was observed in patients undergoing four cycles of NAC containing epirubicin when compared to patients undergoing four cycles of docetaxel. M = midterm; B = baseline; N = number of patients, min = minimum; max = maximum

*Change between baseline and midterm (M-B)*					
		*CON*		*REV*	
*Factor*	*N*	*Median*	*N*	*Median*	*p-value*
		*(Min, Max)*		*(Min, Max)*	
uPA	39	-79.9 (-1391.0, 2168.2)	42	563.0 (-9843.9, 5260.4)	0.0001
CXCL10 (IP10)	39	-12.9 (-383.9, 26.2)	41	17.7 (-93.8, 161.9)	0.0001
sTNFSF10 (sTRAIL)	39	-5.9 (-12,955.7, 95.9)	41	3.4 (-1113.5, 94.8)	0.0005
suPAR	39	-381.1 (-4845.4, 2106.6)	42	267.5 (-6872.9, 5487.1)	0.0020
CCL8 (MCP-2)	39	-9.3 (-71.1, 14.5)	41	0.5 (-20.7, 32.4)	0.0234
CCL2 (MCP-1)	39	-17.4 (-196.8, 79.9)	41	1.6 (-256.6, 157.8)	0.0548
sTIM-3	39	-6.9 (-14,467.4, 2186.9)	41	71.3 (-460.8, 681.8)	0.0823
sCD27	39	-179.5 (-2115.8, 7743.4)	41	22.9 (-3587.8, 3397.6)	0.0893
CSF-1 (M-CSF)	39	-2.5 (-22.6, 61.1)	41	0.0 (-19.7, 14.0)	0.1178
sPD-L2	39	-148.9 (-1145.6, 2076.9)	41	-57.8 (-1807.2, 1214.8)	0.1224
VEGF-A	39	36.6 (-2937.2, 5025.5)	41	-8.9 (-228.8, 819.9)	0.3681
OPG	39	0.0 (-441.7, 116.2)	41	-0.7 (-31.3, 29.6)	0.3729
sTNFSF12 (TWEAK)	39	-59.0 (-1449.4, 591.2)	41	-39.0 (-291.4, 608.0)	0.4216
CCL11 (Eotaxin-1)	39	-0.4 (-36.7, 45.6)	41	4.9 (-138.1, 35.1)	0.5097

Taken together, these data show that the treatment of BC patients with EC reduces B cells, T cells and several soluble immunomodulatory factors in the peripheral blood. Treatment with D, in contrast, maintains lymphocyte number and function, even an increase of some soluble factors in response to D was observable, suggesting that this treatment might have greater inductive potential on immune signaling.

### Docetaxel and epirubicin induce different forms of cell death

**Fig. 3:**
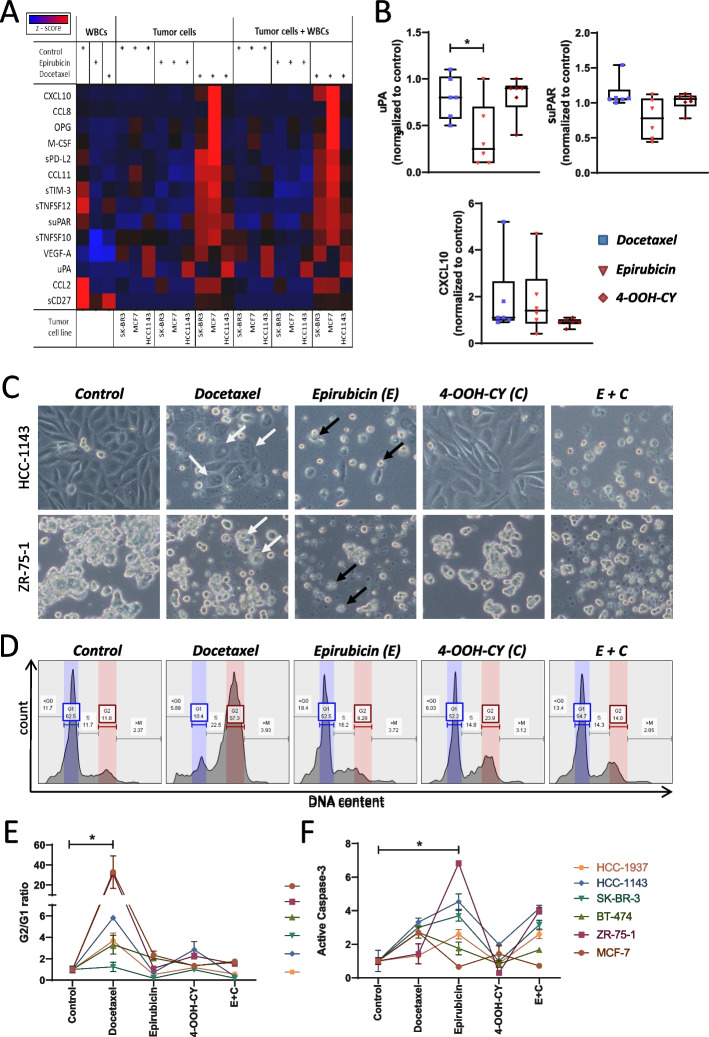
Effect of docetaxel, epirubicin, and cyclophosphamide on in vitro cultures of breast cancer cells. **A** Heat map showing the effect of epirubicin and docetaxel on the release of soluble immune mediators. Epirubicin, docetaxel, or medium as negative control were added to three different breast cancer cell lines (SK-BR3, MCF7, and HCC1143) which were then cultured for 48 h then white blood cells (WBC) were added and the co-culture was cultivated for additional 24 h. The concentration of 14 different immune mediators in the supernatant was determined using a bead array immunoassay. The red colored dots indicate high concentrations of the respective mediator. **B** Release of uPA, suPAR, and CXCL10 from HCC-1937, HCC-1143, SK-BR-3, BT-474, ZR-75–1, and MCF-7 cells in response to docetaxel, epirubicin, and 4-OOH-CY. **C** Phase contrast microscopic images of HCC1143 and ZR-75–1 cells after a 48 h cultivation in the presence of docetaxel, epirubicin, 4-OOH-CY, or a combination of the latter two. The white arrows indicate multinucleated giant cells. The black arrows point to apoptotic cells surrounded by apoptotic bodies. **D** Cell cycle analysis: Cells were treated as described in C and the intracellular DNA was stained with PI. The graph shows the distribution of the fluorescence intensities as assessed by flow cytometry. G1, G2, and S indicate the respective phase of the cell cycle. **E** Summary of the data shown in D. The results are expressed as mean G2/G1 ratio of the respective cell line (± SD, *n* = 3). **F** Quantification of apoptosis. Cells were treated as described in C, then the membrane was permeabilized and stained with antibodies against active caspase 3. The fluorescence was analyzed by flow cytometry. The graph indicates the mean relative distribution normalized to the untreated control (± SD, *n* = 3). The significance was calculated using a one-way ANOWA overall cell lines

To investigate the source of the D-induced immunomodulatory factors we performed in vitro experiments. WBCs, breast cancer cell lines and a combination of both were exposed for 72 h to either epirubicin or docetaxel. PBS served as negative control. Then the above-mentioned 14 factors were measured in the supernatant. The heat map in Fig. 3A gives an overview of the results (for more detailed results see Supplementary Table S4). Docetaxel induced a secretion of many factors including CXCL10, suPAR, and uPA from cancer cell lines, while WBCs remained unresponsive. Epirubicin, in contrast, induced no increase of any of these factors. These results confirm that the immunomodulatory factors, which increased after four cycles of D in vivo, originated from dying tumor cells, rather than immune cells. To confirm that this is a general effect in breast cancer we repeated this experiment with six different breast cancer cell lines (HCC-1937, HCC-1143, SK-BR-3, BT-474, ZR-75–1, and MCF-7) investigating those three parameters, which showed the highest and most significant differential response in the in vivo setting (uPA, suPAR, and CXCL10). These experiments included also a treatment with 4-OOH-CY, which is an active metabolite of cyclophosphamide. Figure 3B shows that epirubicin induced less secretion of uPA and suPAR compared to docetaxel, whereas 4-OOH-CY had no effect. The secretion of CXCL10 varied strongly between cell lines and there was no clear difference between epirubicin-treated and docetaxel-treated cells.

To investigate the respective cell death pathways, we compared the cytotoxic effects of docetaxel, epirubicin and 4-OOH-CY on six breast cancer cell lines. Figure 3C shows examples of microscopic images of two BC cell lines exposed for 48 h to these compounds. Docetaxel-treated cells increased in size forming multinucleated giant cells with accumulated intracellular vacuole. In contrast, epirubicin-treated cells showed classical apoptotic morphological features such as cell shrinkage and numerous apoptotic extracellular vesicles, which are known to be released during the process of apoptotic blebbing. 4-OOH-CY did not affect the cell viability. Next we performed cell cycle measurements (see Fig. 3D). Docetaxel induced an accumulation of cells in G2 phase and a strong decrease of cells in G1. In contrast, epirubicin-treatment caused an increase of cells in G1 and the formation of sub-G1 cells, which reflects a DNA laddering during apoptosis. 4-OOH-CY as a single agent did not affect the cell cycle and showed no additive effect in combination with epirubicin. Figure 3E summarizes the results of the cell cycle measurements. Docetaxel induced a significant increase of the G2/G1 ratio in all six cell lines. All other treatments had no significant effect as compared to control. To further specify the induction of apoptosis, an active caspase-3 assay was performed. Epirubicin-treated cells showed a significantly higher activation of caspase-3 as compared to control (see Fig. 3F). Docetaxel-treated cell showed also some increase, but to a lower extend. 4OOH-CY had no effect. Taken together these results confirm that epirubicin and docetaxel differ in their induction of cell death in breast cancer cell lines, where docetaxel-treatment is associated with the release of various immunomodulatory mediators similar as observed above in vivo.

## Discussion

Neoadjuvant anthracyclines and taxanes still represent one standard-of-care therapy option in women with early, high-risk or locally advanced breast cancer [24, 25]. In TNBC patients, further substantial benefits can be achieved by adding immune checkpoint inhibitors either in the neoadjuvant setting [11, 13, 26] or even in the locally advanced or metastatic setting [27]. Since the combination of chemotherapy with immunotherapy is more and more common, the question arises if there are agents that should be preferentially combined while other combinations may cause mutual interference, potentially impeding immune modulating characteristics of a particular agent. The study design of the ABCSG-34 trial allowed for a comparison of the conventional (EC followed by D) versus reverse (D followed by EC) NAC scheme with respect to in vivo immune effects. It enabled a profound investigation of the distinct effects of each chemotherapeutic agent on its own during the initial 4 cycles of therapy. Although Bartsch et al. overall found no differences regarding clinical and pathological response to NAC between patients undergoing conventional or reverse NAC [16], the here presented study revealed that EC and D differ fundamentally in their effects on lymphocytes and on immunomodulatory molecules in the circulation. Patients on upfront EC developed lymphopenia with CD19^+^ B cells decreasing to less than 10% of respective baseline levels, whereas D had no ablating effects. This effect was long-lasting since the suppression persisted even after the following four cycles of D. A similar durable inhibitory impact of EC on B cells was observed in a study by Verma et al. [28]. A major depletion of B cells was observed, dropping to a median of 5.4% of pre-chemotherapy levels. After 9 months, a partial recovery of B and CD4^+^ T cells was observed although their levels remained significantly lower than before chemotherapy. Other trials showed similar effects of EC and confirmed a B cell loss of over 90% after administration of EC in TNBC patients. Additionally, they reported decreased numbers of natural killer cells and CD4^+^ T lymphocytes to 50% while the frequency of CD8^+^ T cells was less affected [29]. Similarly, we observed that T cells were not as sensitive to EC as B cells. CD3^+^ T cells decreased after four cycles of EC by only 50%. EC administered after D had no detectable effect on CD3^+^ T cells. The latter probably has to do with the large variation observed in T cells after treatment with D, which may mask any weak EC effect. Taken together, EC administration entailed a strong and long-lasting suppressive effect on B cells and a more transient effect on T cells while D had only a minor impact on lymphocytes and their subtypes.

In the here presented study the stimulatory effects of D on soluble immunomodulatory biomarkers were clearly observable in vivo as well as in vitro. EC conversely led to a decrease of plasma levels of diverse soluble immunomodulatory molecules such as CXCL-10, uPA, suPAR, MCP-1, MCP-2 and Tweak in vivo and to decreasing levels in our in vitro experiments. Of note, we cannot clarify whether the effects of the initial four cycles of EC can be attributed either to E or C or to the combination of both. The alkylating agent C is a well-known immunosuppressant, commonly used to prevent graft rejection or to treat several chronic autoimmune disorders [30]. To adequately assess the cytotoxic effect of C by itself, it was investigated separately in the in vitro experiments, where we used concentrations of E, C and D, which were in the lower range of published plasma peak concentration: 2 µM epirubicin (range: 2–6 µM) [29], 15 µM active cyclophosphamide metabolite 4-OOH-CY cyclophosphamide (range: 15–36 µM) [30], and 1 µM docetaxel (range: 1–6 µM)s [31]. Although 4-OOH-CY is known to induce caspase-9 dependent apoptosis in 9L gliosarcoma cells at a similar concentration as it was used here [32], no cytotoxic effect was observable in any tested cell line when compared to E and D. This might be explained by the finding that the apoptosis-inducing effect of 4-OOH-CY varies strongly between cell types [33]. Since all of the six tested BC cell lines reacted similarly to E as well as to D we concluded that the observed effects were independent of the BC subtype and may affect other solid tumors in an analogous way. Even though docetaxel and epirubicin have been reported to ultimately lead to apoptosis as well [32, 34, 35], the pathway to this differs substantially. Docetaxel prevents microtubule depolymerisation [31], whereas epirubicin stabilizes the topoisomerase II DNA complex [36]. The group of Seamus Martin showed that dying cells release in the very early phase of apoptosis various chemokines which serve as “find-me” signals for immune cells for the clearance of the cell debris [37]. This involves apoptosis-related proteins such as RIPK1 and IAPs and depends on the apoptosis-inducing agent. Similarly, we found recently that apoptotic colorectal cancer cells secrete CXCL1, CXCL5, and CXCL8 in response to chemotherapeutic drugs such as oxaliplatin, 5-FU, or bortezomib [23]. The increased release of uPA, suPAR, and CXCL10 immunomodulatory factors by D-treated breast cancer cell lines observed here, might be related to a similar mechanism. From the data shown here, we cannot conclude whether and how these immunomodulatory factors affect immune cells in the tumor tissue. A breast cancer model in mice with a humanized immune system would be necessary for this purpose. However, this would go far beyond the scope of the present study.

In a recently published work Lopes et al. stated that the immune response is key to successful neoadjuvant chemotherapy [38]. In their study they observed higher levels of plasma immune mediators like VEGF-A, GM-CSF and IL-2 at baseline in TNBC patients responding to NAC compared with non-responders. They further observed that systemic inflammatory cytokines were positively correlated with levels of TILs in patients achieving pCR. The predictive relevance of lymphocytes in the tumor microenvironment, however, is well described in the literature [39–41]. Herrero-Vicent et al. demonstrated that TNBC patients with a so-called lymphocyte-predominant breast cancer receiving NAC with anthracyclines and taxanes had higher pCR rates (88%) than those with a non-lymphocyte-predominant BC (9%) and thus suggested that TILs could be routinely used as biomarker [42]. The predictive value of TILs irrespective of breast cancer subtype was confirmed in an analysis performed by the German Breast Cancer Group, where high levels of TILs predicted for higher pCR rates in patients with luminal, HER2 + and triple-negative disease [43]. In our study cohort, including HR + /HER2- and TNBC patients, we found that patients with high TILs at baseline as well as patients with increasing TILs during NAC had a better response to NAC. This agrees with observations in other studies. A recent systematic review and meta-analysis confirmed that higher levels of TILs after NAC correlated with significantly improved recurrence-free, metastasis-free and event-free survival in TNBC patients [44].

Limitations of our study were the retrospective nature of the study, the lack of long-term outcomes due to currently missing follow-up data as well as the division into subgroups depending on the NAC scheme, which led to a comparison of two smaller groups. With the intention to investigate the distinct effects of EC and D, ABCSG-34 patients receiving neoadjuvant AI therapy as well as all vaccinated patients were excluded. Reasons for this were the low risk profile of patients who were selected for neoadjuvant AI therapy in the ABCSG-34 trial and the unpredictable effect of the Mucin-1 cancer vaccine on circulating biomarkers and on sTILs or iTILs. Further, it has to be pointed out that the selection of immunomodulatory biomarkers was based on preliminary data from ABCSG-34 patients who received neoadjuvant AI therapy (see Supplementary Figure S3) although those patients were excluded from further analysis in the here presented work. We assume that a potential bias might be neglectable since this preliminary data was used to define a large number of measurable parameters in patients with invasive but low risk BC. Regarding the predictive value of sTILs and iTILs during NAC, it must be emphasized that the small number of cases definitely influenced the power of this analysis with odds ratios only marginally higher than 1. Thus, the results regarding the predictive value of NAC-induced changes in sTILs and iTILs have to be interpreted with caution. Nevertheless, the statistical significance of these results indicates that EC might affect lymphocytes in a different way than D does.

As chemotherapy, either in neoadjuvant or adjuvant manner, is frequently faced with tumor regrowth or drug resistance, a combination with immunotherapy seems promising. There is evidence that the form of cell death induction plays a pivotal role in the level of anti-tumor immune response. In the here presented study, a strong inhibitory effect of EC on lymphocytes was observed. This may counteract the stimulatory effect of immunotherapies, and thus, upfront taxane-therapy may be the preferred approach for trials of immunotherapeutic approaches in early-stage breast cancer.

### Supplementary Information

**Additional file 1.** 

## Data Availability

The dataset used is not publicly available as it may contain information that would compromise patient consent. Please contact the corresponding author for more information. The published results of the dataset from the randomized, prospective ABCSG-34 trial conducted by the Austrian Breast Cancer and Colorectal Study Group (ABCSG) are online available (https://doi.org/10.1016/j.ejca.2020.03.018).

## References

[1] Ferlay J, Soerjomataram I, Dikshit R, Eser S, Mathers C, Rebelo M, et al. Cancer incidence and mortality worldwide: sources, methods and major patterns in GLOBOCAN 2012. Int J cancer. 2015;136:E359–86. Available from: http://www.ncbi.nlm.nih.gov/pubmed/25220842 10.1002/ijc.29210 25220842

[2] Senovilla L, Vacchelli E, Galon J, Adjemian S, Eggermont A, Fridman WH, et al. Trial watch: Prognostic and predictive value of the immune infiltrate in cancer. Oncoimmunology. 2012;1:1323–43. Available from: http://www.tandfonline.com/doi/abs/10.4161/onci.22009 10.4161/onci.22009 PMC3518505 23243596

[3] Cunha LL, Morari EC, Guihen ACT, Razolli D, Gerhard R, Nonogaki S, et al. Infiltration of a mixture of different immune cells may be related to molecular profile of differentiated thyroid cancer. Endocr Relat Cancer. 2012;19:L31–6. Available from: http://www.ncbi.nlm.nih.gov/pubmed/22461634 10.1530/ERC-11-0285 22461634

[4] Mahmoud SMA, Paish EC, Powe DG, Macmillan RD, Grainge MJ, Lee AHS, et al. Tumor-infiltrating CD8+ lymphocytes predict clinical outcome in breast cancer. J Clin Oncol. 2011;29:1949–55. Available from: http://www.ncbi.nlm.nih.gov/pubmed/21483002 10.1200/JCO.2010.30.5037 21483002

[5] Kawai O, Ishii G, Kubota K, Murata Y, Naito Y, Mizuno T, et al. Predominant infiltration of macrophages and CD8(+) T Cells in cancer nests is a significant predictor of survival in stage IV nonsmall cell lung cancer. Cancer [Internet]. 2008;113:1387–95. Available from: http://www.ncbi.nlm.nih.gov/pubmed/18671239 10.1002/cncr.23712 18671239

[6] Demaria S, Volm MD, Shapiro RL, Yee HT, Oratz R, Formenti SC, et al. Development of tumor-infiltrating lymphocytes in breast cancer after neoadjuvant paclitaxel chemotherapy. Clin Cancer Res [Internet]. 2001;7:3025–30. Available from: http://www.ncbi.nlm.nih.gov/pubmed/11595690 11595690

[7] Fridman WH, Zitvogel L, Sautès–Fridman C, Kroemer G. The immune contexture in cancer prognosis and treatment. Nat Rev Clin Oncol [Internet]. 2017;14:717–34. Available from: http://www.nature.com/articles/nrclinonc.2017.101 10.1038/nrclinonc.2017.101 28741618

[8] Wu J, Waxman DJ. Immunogenic chemotherapy: Dose and schedule dependence and combination with immunotherapy. Cancer Lett. 2018;419:210–21. Available from: http://www.ncbi.nlm.nih.gov/pubmed/29414305 10.1016/j.canlet.2018.01.050 PMC5818299 29414305

[9] Sprooten J, Laureano RS, Vanmeerbeek I, Govaerts J, Naulaerts S, Borras DM, et al. Trial watch: chemotherapy-induced immunogenic cell death in oncology. Oncoimmunology. 2023;12:2219591. Available from: http://www.ncbi.nlm.nih.gov/pubmed/37284695 10.1080/2162402X.2023.2219591 PMC10240992 37284695

[10] Cao Y, Chen C, Tao Y, Lin W, Wang P. Immunotherapy for Triple-Negative Breast Cancer. Pharmaceutics. 2021;13. Available from: http://www.ncbi.nlm.nih.gov/pubmed/34959285 10.3390/pharmaceutics13122003 PMC8705248 34959285

[11] Schmid P, Dent R, O’Shaughnessy J. Pembrolizumab for Early Triple-Negative Breast Cancer. Reply. N Engl J Med. 2020;382:e108. Available from: http://www.ncbi.nlm.nih.gov/pubmed/32579835 10.1056/NEJMc2006684 32579835

[12] Gianni L, Huang CS, Egle D, Bermejo B, Zamagni C, Thill M, et al. Pathologic complete response (pCR) to neoadjuvant treatment with or without atezolizumab in triple-negative, early high-risk and locally advanced breast cancer: NeoTRIP Michelangelo randomized study. Ann Oncol Off J Eur Soc Med Oncol. 2022;33:534–43. Available from: http://www.ncbi.nlm.nih.gov/pubmed/35182721 10.1016/j.annonc.2022.02.004 35182721

[13] Loibl S, Untch M, Burchardi N, Huober J, Sinn B V, Blohmer J-U, et al. A randomised phase II study investigating durvalumab in addition to an anthracycline taxane-based neoadjuvant therapy in early triple-negative breast cancer: clinical results and biomarker analysis of GeparNuevo study. Ann Oncol Off J Eur Soc Med Oncol. 2019;30:1279–88. Available from: http://www.ncbi.nlm.nih.gov/pubmed/31095287 10.1093/annonc/mdz158 31095287

[14] Schmid P, Rugo HS, Adams S, Schneeweiss A, Barrios CH, Iwata H, et al. Atezolizumab plus nab-paclitaxel as first-line treatment for unresectable, locally advanced or metastatic triple-negative breast cancer (IMpassion130): updated efficacy results from a randomised, double-blind, placebo-controlled, phase 3 trial. Lancet Oncol. 2020;21:44–59. Available from: http://www.ncbi.nlm.nih.gov/pubmed/31786121 10.1016/S1470-2045(19)30689-8 31786121

[15] Singer CF, Pfeiler G, Hubalek M, Bartsch R, Stöger H, Pichler A, et al. Efficacy and safety of the therapeutic cancer vaccine tecemotide (L-BLP25) in early breast cancer: Results from a prospective, randomised, neoadjuvant phase II study (ABCSG 34). Eur J Cancer. 2020;132:43–52. Available from: http://www.ncbi.nlm.nih.gov/pubmed/32325419 10.1016/j.ejca.2020.03.018 32325419

[16] Bartsch R, Singer CF, Pfeiler G, Hubalek M, Stoeger H, Pichler A, et al. Conventional versus reverse sequence of neoadjuvant epirubicin/cyclophosphamide and docetaxel: sequencing results from ABCSG-34. Br J Cancer. 2021;124:1795–802. Available from: http://www.ncbi.nlm.nih.gov/pubmed/33762716 10.1038/s41416-021-01284-2 PMC8144560 33762716

[17] Symmans WF, Wei C, Gould R, Yu X, Zhang Y, Liu M, et al. Long-Term Prognostic Risk After Neoadjuvant Chemotherapy Associated With Residual Cancer Burden and Breast Cancer Subtype. J Clin Oncol. 2017;35:1049–60. Available from: http://www.ncbi.nlm.nih.gov/pubmed/28135148 10.1200/JCO.2015.63.1010 PMC5455352 28135148

[18] Salgado R, Denkert C, Demaria S, Sirtaine N, Klauschen F, Pruneri G, et al. The evaluation of tumor-infiltrating lymphocytes (TILs) in breast cancer: recommendations by an International TILs Working Group 2014. Ann Oncol Off J Eur Soc Med Oncol. 2015;26:259–71. Available from: http://www.ncbi.nlm.nih.gov/pubmed/25214542 10.1093/annonc/mdu450 PMC6267863 25214542

[19] Denkert C, Wienert S, Poterie A, Loibl S, Budczies J, Badve S, et al. Standardized evaluation of tumor-infiltrating lymphocytes in breast cancer: results of the ring studies of the international immuno-oncology biomarker working group. Mod Pathol. 2016;29:1155–64. Available from: http://www.ncbi.nlm.nih.gov/pubmed/27363491 10.1038/modpathol.2016.109 27363491

[20] Prat A, Karginova O, Parker JS, Fan C, He X, Bixby L, et al. Characterization of cell lines derived from breast cancers and normal mammary tissues for the study of the intrinsic molecular subtypes. Breast Cancer Res Treat. 2013;142:237–55. Available from: http://www.ncbi.nlm.nih.gov/pubmed/24162158 10.1007/s10549-013-2743-3 PMC3832776 24162158

[21] Dai X, Cheng H, Bai Z, Li J. Breast Cancer Cell Line Classification and Its Relevance with Breast Tumor Subtyping. J Cancer. 2017;8:3131–41. Available from: http://www.ncbi.nlm.nih.gov/pubmed/29158785 10.7150/jca.18457 PMC5665029 29158785

[22] Liang YY, Schwarzinger I, Simonitsch-Klupp I, Agis H, Oehler R. Impaired efferocytosis by monocytes in multiple myeloma. Oncol Lett. 2018;16:409–16. Available from: http://www.spandidos-publications.com/10.3892/ol.2018.8620 10.3892/ol.2018.8620 PMC6006325 29928429

[23] Schimek V, Strasser K, Beer A, Göber S, Walterskirchen N, Brostjan C, et al. Tumour cell apoptosis modulates the colorectal cancer immune microenvironment via interleukin-8-dependent neutrophil recruitment. Cell Death Dis. 2022;13:113. Available from: 10.1038/s41419-022-04585-3 10.1038/s41419-022-04585-3 PMC8816934 35121727

[24] Fisher B, Brown A, Mamounas E, Wieand S, Robidoux A, Margolese RG, et al. Effect of preoperative chemotherapy on local-regional disease in women with operable breast cancer: findings from National Surgical Adjuvant Breast and Bowel Project B-18. J Clin Oncol. 1997;15:2483–93. Available from: http://www.ncbi.nlm.nih.gov/pubmed/9215816 10.1200/JCO.1997.15.7.2483 9215816

[25] Lucas MW, Kelly CM. Optimal Choice of Neoadjuvant Chemotherapy for HER2-Negative Breast Cancer: Clinical Insights. Cancer Manag Res. 2022;14:2493–506. Available from: http://www.ncbi.nlm.nih.gov/pubmed/35999966 10.2147/CMAR.S341466 PMC9393016 35999966

[26] Nanda R, Liu MC, Yau C, Shatsky R, Pusztai L, Wallace A, et al. Effect of Pembrolizumab Plus Neoadjuvant Chemotherapy on Pathologic Complete Response in Women With Early-Stage Breast Cancer: An Analysis of the Ongoing Phase 2 Adaptively Randomized I-SPY2 Trial. JAMA Oncol. 2020;6:676–84. Available from: http://www.ncbi.nlm.nih.gov/pubmed/32053137 10.1001/jamaoncol.2019.6650 PMC7058271 32053137

[27] Schmid P, Adams S, Rugo HS, Schneeweiss A, Barrios CH, Iwata H, et al. Atezolizumab and Nab-Paclitaxel in Advanced Triple-Negative Breast Cancer. N Engl J Med. 2018;379:2108–21. Available from: http://www.ncbi.nlm.nih.gov/pubmed/30345906 10.1056/NEJMoa1809615 30345906

[28] Verma R, Foster RE, Horgan K, Mounsey K, Nixon H, Smalle N, et al. Lymphocyte depletion and repopulation after chemotherapy for primary breast cancer. Breast Cancer Res. 2016;18:10. Available from: http://breast-cancer-research.biomedcentral.com/articles/10.1186/s13058-015-0669-x 10.1186/s13058-015-0669-x PMC4727393 26810608

[29] Massa C, Karn T, Denkert C, Schneeweiss A, Hanusch C, Blohmer J-U, et al. Differential effect on different immune subsets of neoadjuvant chemotherapy in patients with TNBC. J Immunother cancer. 2020;8:e001261. Available from: http://www.ncbi.nlm.nih.gov/pubmed/33199511 10.1136/jitc-2020-001261 PMC7670944 33199511

[30] Ahlmann M, Hempel G. The effect of cyclophosphamide on the immune system: implications for clinical cancer therapy. Cancer Chemother. Pharmacol. Springer Verlag; 2016 [cited 2021 Apr 21]. p. 661–71. Available from: https://pubmed.ncbi.nlm.nih.gov/27646791/10.1007/s00280-016-3152-1 27646791

[31] Clarke SJ, Rivory LP. Clinical pharmacokinetics of docetaxel. Clin Pharmacokinet. 1999/03/27. 1999;36:99–114. Available from: http://www.ncbi.nlm.nih.gov/pubmed/10092957 10.2165/00003088-199936020-00002 10092957

[32] Schwartz PS, Waxman DJ. Cyclophosphamide induces caspase 9-dependent apoptosis in 9L tumor cells. Mol Pharmacol. 2001;60:1268–79. Available from: http://www.ncbi.nlm.nih.gov/pubmed/11723234 10.1124/mol.60.6.1268 11723234

[33] Chow LWC, Loo WTY. The differential effects of cyclophosphamide, epirubicin and 5-fluorouracil on apoptotic marker (CPP-32), pro-apoptotic protein (p21(WAF-1)) and anti-apoptotic protein (bcl-2) in breast cancer cells. Breast Cancer Res Treat. 2003;80:239–44. Available from: http://www.ncbi.nlm.nih.gov/pubmed/14503796 10.1023/A:1024995202135 14503796

[34] Caraglia M, Giuberti G, Marra M, Di Gennaro E, Facchini G, Caponigro F, et al. Docetaxel induces p53-dependent apoptosis and synergizes with farnesyl transferase inhibitor r115777 in human epithelial cancer cells. Front Biosci. 2005;10:2566–75. Available from: http://www.ncbi.nlm.nih.gov/pubmed/15970518 10.2741/1720 15970518

[35] Huang T-C, Chiu P-R, Chang W-T, Hsieh B-S, Huang Y-C, Cheng H-L, et al. Epirubicin induces apoptosis in osteoblasts through death-receptor and mitochondrial pathways. Apoptosis. 2018;23:226–36. Available from: http://www.ncbi.nlm.nih.gov/pubmed/29468482 10.1007/s10495-018-1450-2 29468482

[36] Mouridsen HT, Alfthan C, Bastholt L, Bergh J, Dalmark M, Eksborg S, et al. Current status of epirubicin (Farmorubicin) in the treatment of solid tumours. Acta Oncol. 1990;29:257–85. Available from: http://www.ncbi.nlm.nih.gov/pubmed/2194531 10.3109/02841869009089998 2194531

[37] Cullen SP, Henry CM, Kearney CJ, Logue SE, Feoktistova M, Tynan GA (2013). Fas/CD95-induced chemokines can serve as “find-me” signals for apoptotic cells. Mol Cell.

[38] Lopes AD, Galdino NAL, Figueiredo AB, Brianese RC, Morais KLP, De Brot M, et al. Systemic immune mediators reflect tumour-infiltrating lymphocyte intensity and predict therapeutic response in triple-negative breast cancer. Immunology. 2023;169:229–41. Available from: http://www.ncbi.nlm.nih.gov/pubmed/36703241 10.1111/imm.13627 36703241

[39] Abuhadra N, Sun R, Yam C, Rauch GM, Ding Q, Lim B, et al. Predictive Roles of Baseline Stromal Tumor-Infiltrating Lymphocytes and Ki-67 in Pathologic Complete Response in an Early-Stage Triple-Negative Breast Cancer Prospective Trial. Cancers (Basel). 2023;15. Available from: http://www.ncbi.nlm.nih.gov/pubmed/37444385 10.3390/cancers15133275 PMC10339918 37444385

[40] Thagaard J, Broeckx G, Page DB, Jahangir CA, Verbandt S, Kos Z, et al. Pitfalls in machine learning-based assessment of tumor-infiltrating lymphocytes in breast cancer: A report of the International Immuno-Oncology Biomarker Working group on Breast Cancer. J Pathol. 2023;260:498–513. Available from: http://www.ncbi.nlm.nih.gov/pubmed/37608772 10.1002/path.6155 PMC10518802 37608772

[41] Ochi T, Bianchini G, Ando M, Nozaki F, Kobayashi D, Criscitiello C, et al. Predictive and prognostic value of stromal tumour-infiltrating lymphocytes before and after neoadjuvant therapy in triple negative and HER2-positive breast cancer. Eur J Cancer. 2019;118:41–8. Available from: http://www.ncbi.nlm.nih.gov/pubmed/31302586 10.1016/j.ejca.2019.05.014 31302586

[42] Herrero-Vicent C, Guerrero A, Gavilá J, Gozalbo F, Hernández A, Sandiego S, et al. Predictive and prognostic impact of tumour-infiltrating lymphocytes in triple-negative breast cancer treated with neoadjuvant chemotherapy. Ecancermedicalscience. 2017;11:759. Available from: http://www.ncbi.nlm.nih.gov/pubmed/28900472 10.3332/ecancer.2017.759 PMC5574654 28900472

[43] Denkert C, von Minckwitz G, Darb-Esfahani S, Lederer B, Heppner BI, Weber KE, et al. Tumour-infiltrating lymphocytes and prognosis in different subtypes of breast cancer: a pooled analysis of 3771 patients treated with neoadjuvant therapy. Lancet Oncol. 2018;19:40–50. Available from: http://www.ncbi.nlm.nih.gov/pubmed/29233559 10.1016/S1470-2045(17)30904-X 29233559

[44] Cao B, Zhang Z, Wang C, Lv X. Prognostic relevance of tumor‑infiltrating lymphocytes in residual tumor tissue from patients with triple‑negative breast cancer following neoadjuvant chemotherapy: A systematic review and meta‑analysis. Oncol Lett. 2023;26:441. Available from: http://www.spandidos-publications.com/10.3892/ol.2023.14028 10.3892/ol.2023.14028 PMC10472026 37664648

