# Supplementary material for: Differential immunomodulatory effects of epirubicin/cyclophosphamide and docetaxel in breast cancer patients
Source: J Exp Clin Cancer Res. 2023 Nov 14;42:300. doi: 10.1186/s13046-023-02876-x (PMC10644559; doi:10.1186/s13046-023-02876-x)
Supplement: Supplementary file 1 — Additional file 1. [file 13046_2023_2876_MOESM1_ESM.pptx]

## Slide 1
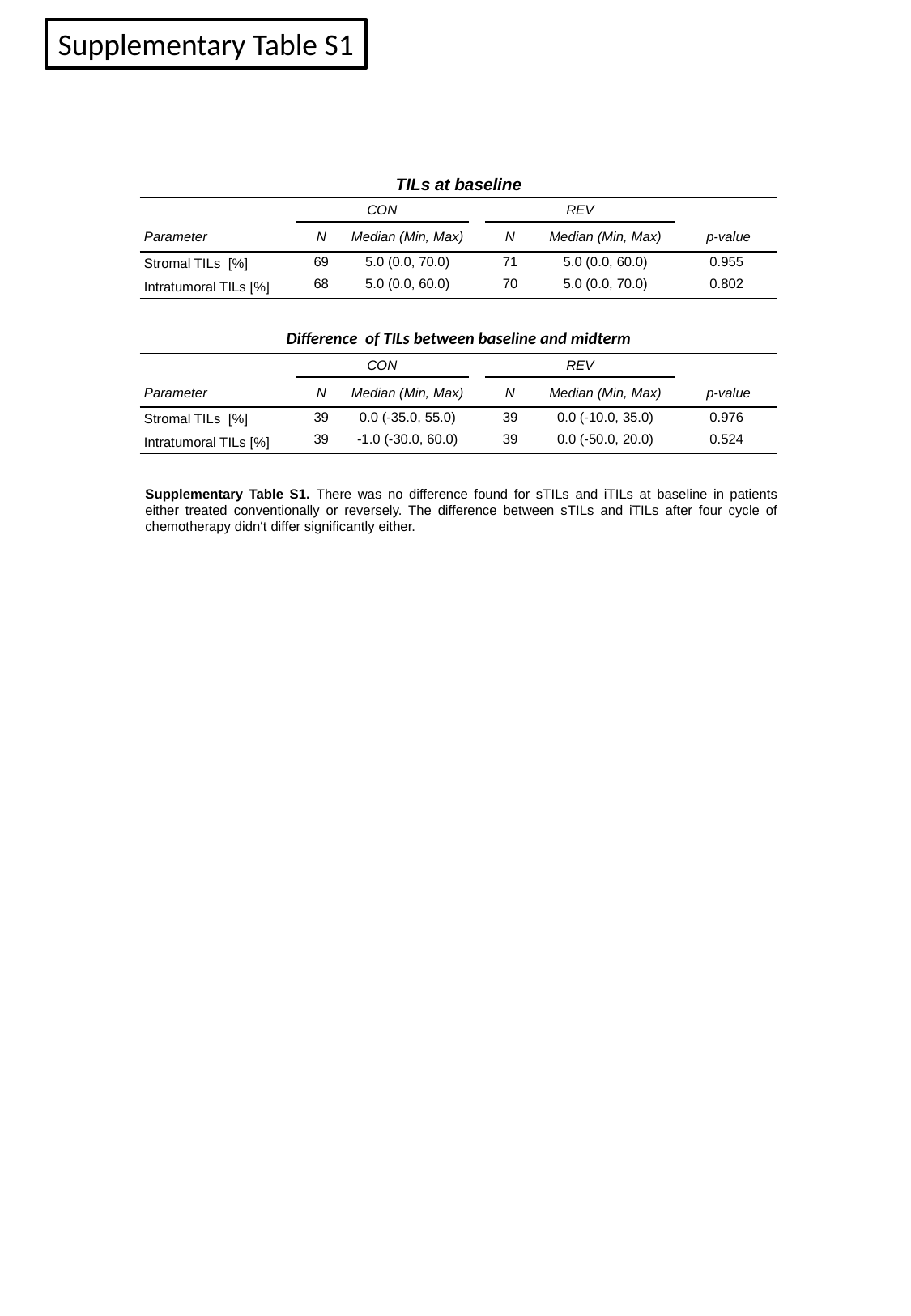

Supplementary Table S1
| TILs at baseline | | | | | | |
| --- | --- | --- | --- | --- | --- | --- |
| | CON | | | REV | | |
| Parameter | N | Median (Min, Max) | | N | Median (Min, Max) | p-value |
| Stromal TILs [%] | 69 | 5.0 (0.0, 70.0) | | 71 | 5.0 (0.0, 60.0) | 0.955 |
| Intratumoral TILs [%] | 68 | 5.0 (0.0, 60.0) | | 70 | 5.0 (0.0, 70.0) | 0.802 |
| Difference of TILs between baseline and midterm | | | | | | |
| --- | --- | --- | --- | --- | --- | --- |
| | CON | | | REV | | |
| Parameter | N | Median (Min, Max) | | N | Median (Min, Max) | p-value |
| Stromal TILs [%] | 39 | 0.0 (-35.0, 55.0) | | 39 | 0.0 (-10.0, 35.0) | 0.976 |
| Intratumoral TILs [%] | 39 | -1.0 (-30.0, 60.0) | | 39 | 0.0 (-50.0, 20.0) | 0.524 |
Supplementary Table S1. There was no difference found for sTILs and iTILs at baseline in patients either treated conventionally or reversely. The difference between sTILs and iTILs after four cycle of chemotherapy didn‘t differ significantly either.

## Slide 2
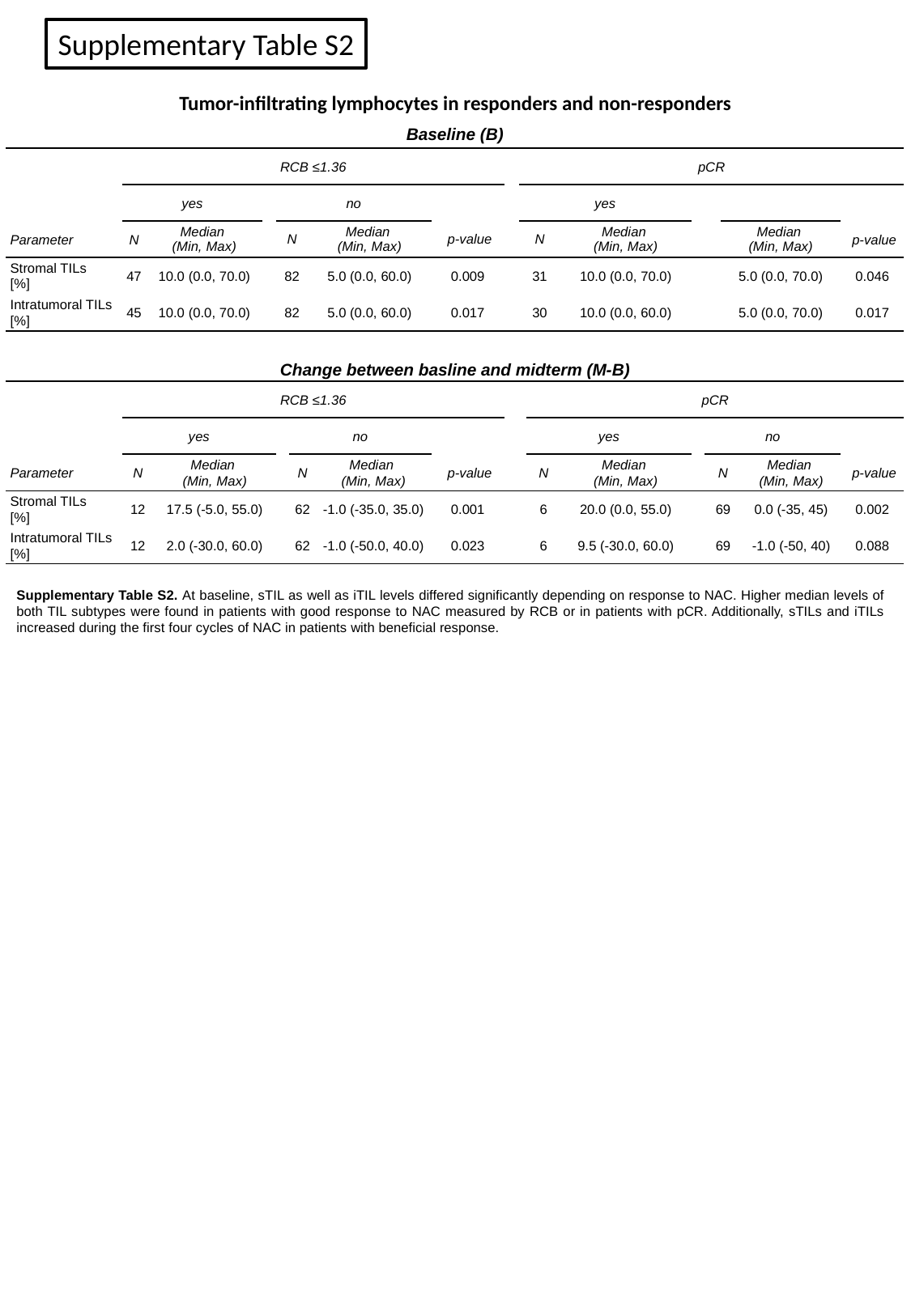

Supplementary Table S2
Tumor-infiltrating lymphocytes in responders and non-responders
| Baseline (B) | | | | | | | | | | | | | | | | | | |
| --- | --- | --- | --- | --- | --- | --- | --- | --- | --- | --- | --- | --- | --- | --- | --- | --- | --- | --- |
| | RCB ≤1.36 | | | | | | | | | | pCR | | | | | | | |
| | yes | | | | no | | | | | | yes | | | | | | | |
| Parameter | N | Median (Min, Max) | | | N | | Median (Min, Max) | | p-value | | N | | Median (Min, Max) | | | Median (Min, Max) | | p-value |
| Stromal TILs [%] | 47 | 10.0 (0.0, 70.0) | | | 82 | | 5.0 (0.0, 60.0) | | 0.009 | | 31 | | 10.0 (0.0, 70.0) | | | 5.0 (0.0, 70.0) | | 0.046 |
| Intratumoral TILs [%] | 45 | 10.0 (0.0, 70.0) | | | 82 | | 5.0 (0.0, 60.0) | | 0.017 | | 30 | | 10.0 (0.0, 60.0) | | | 5.0 (0.0, 70.0) | | 0.017 |
| | | | | | | | | | | | | | | | | | | |
| Change between basline and midterm (M-B) | | | | | | | | | | | | | | | | | | |
| | RCB ≤1.36 | | | | | | | | | | | pCR | | | | | | |
| | yes | | | | | no | | | | | | yes | | | no | | | |
| Parameter | N | | Median (Min, Max) | | | N | | Median (Min, Max) | p-value | | | N | Median (Min, Max) | | N | | Median (Min, Max) | p-value |
| Stromal TILs [%] | 12 | | 17.5 (-5.0, 55.0) | | | 62 | | -1.0 (-35.0, 35.0) | 0.001 | | | 6 | 20.0 (0.0, 55.0) | | 69 | | 0.0 (-35, 45) | 0.002 |
| Intratumoral TILs [%] | 12 | | 2.0 (-30.0, 60.0) | | | 62 | | -1.0 (-50.0, 40.0) | 0.023 | | | 6 | 9.5 (-30.0, 60.0) | | 69 | | -1.0 (-50, 40) | 0.088 |
Supplementary Table S2. At baseline, sTIL as well as iTIL levels differed significantly depending on response to NAC. Higher median levels of both TIL subtypes were found in patients with good response to NAC measured by RCB or in patients with pCR. Additionally, sTILs and iTILs increased during the first four cycles of NAC in patients with beneficial response.

## Slide 3
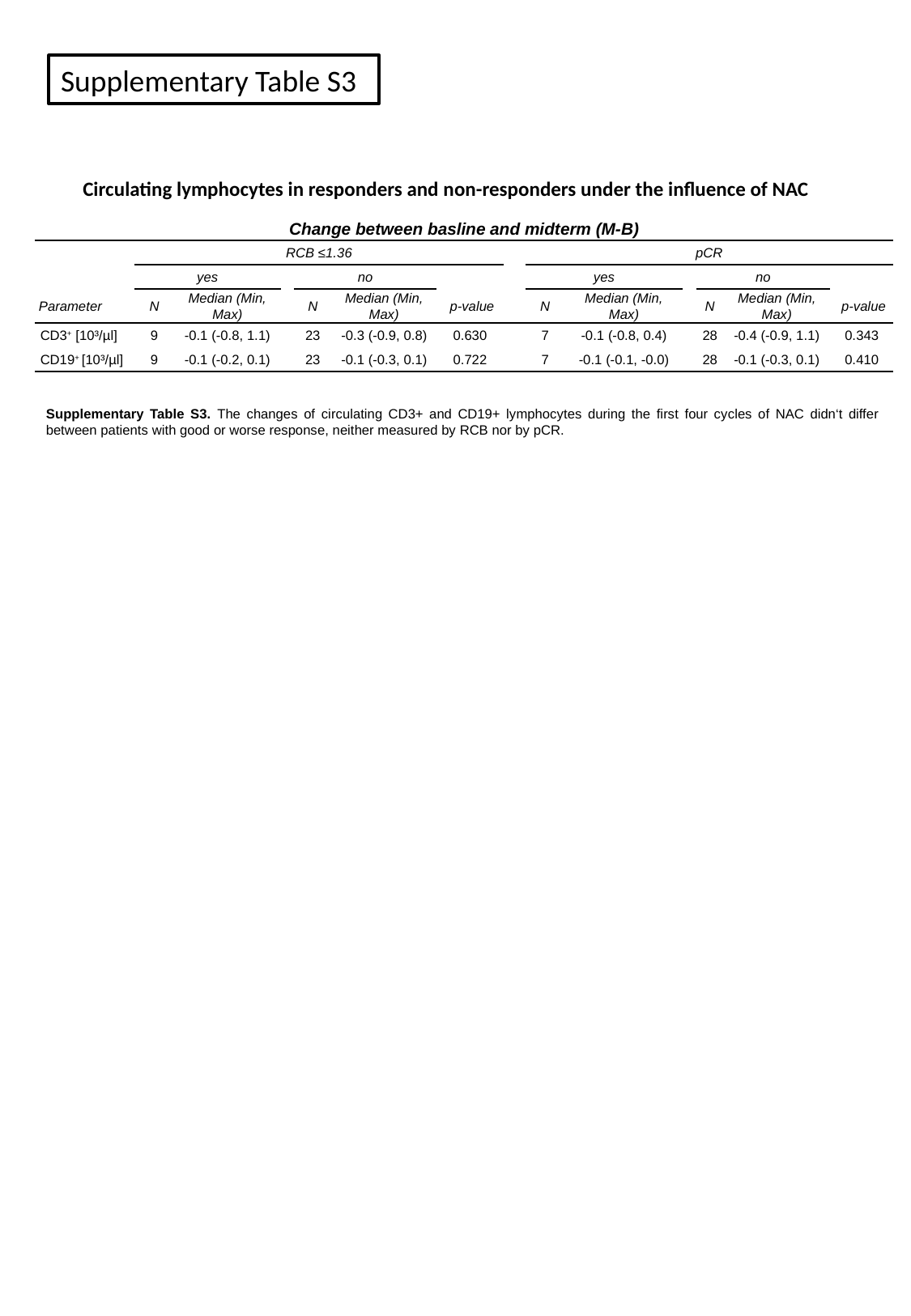

Supplementary Table S3
Circulating lymphocytes in responders and non-responders under the influence of NAC
| Change between basline and midterm (M-B) | | | | | | | | | | | | | |
| --- | --- | --- | --- | --- | --- | --- | --- | --- | --- | --- | --- | --- | --- |
| | RCB ≤1.36 | | | | | | | pCR | | | | | |
| | yes | | | no | | | | yes | | | no | | |
| Parameter | N | Median (Min, Max) | | N | Median (Min, Max) | p-value | | N | Median (Min, Max) | | N | Median (Min, Max) | p-value |
| CD3+ [10³/µl] | 9 | -0.1 (-0.8, 1.1) | | 23 | -0.3 (-0.9, 0.8) | 0.630 | | 7 | -0.1 (-0.8, 0.4) | | 28 | -0.4 (-0.9, 1.1) | 0.343 |
| CD19+ [10³/µl] | 9 | -0.1 (-0.2, 0.1) | | 23 | -0.1 (-0.3, 0.1) | 0.722 | | 7 | -0.1 (-0.1, -0.0) | | 28 | -0.1 (-0.3, 0.1) | 0.410 |
Supplementary Table S3. The changes of circulating CD3+ and CD19+ lymphocytes during the first four cycles of NAC didn‘t differ between patients with good or worse response, neither measured by RCB nor by pCR.

## Slide 4
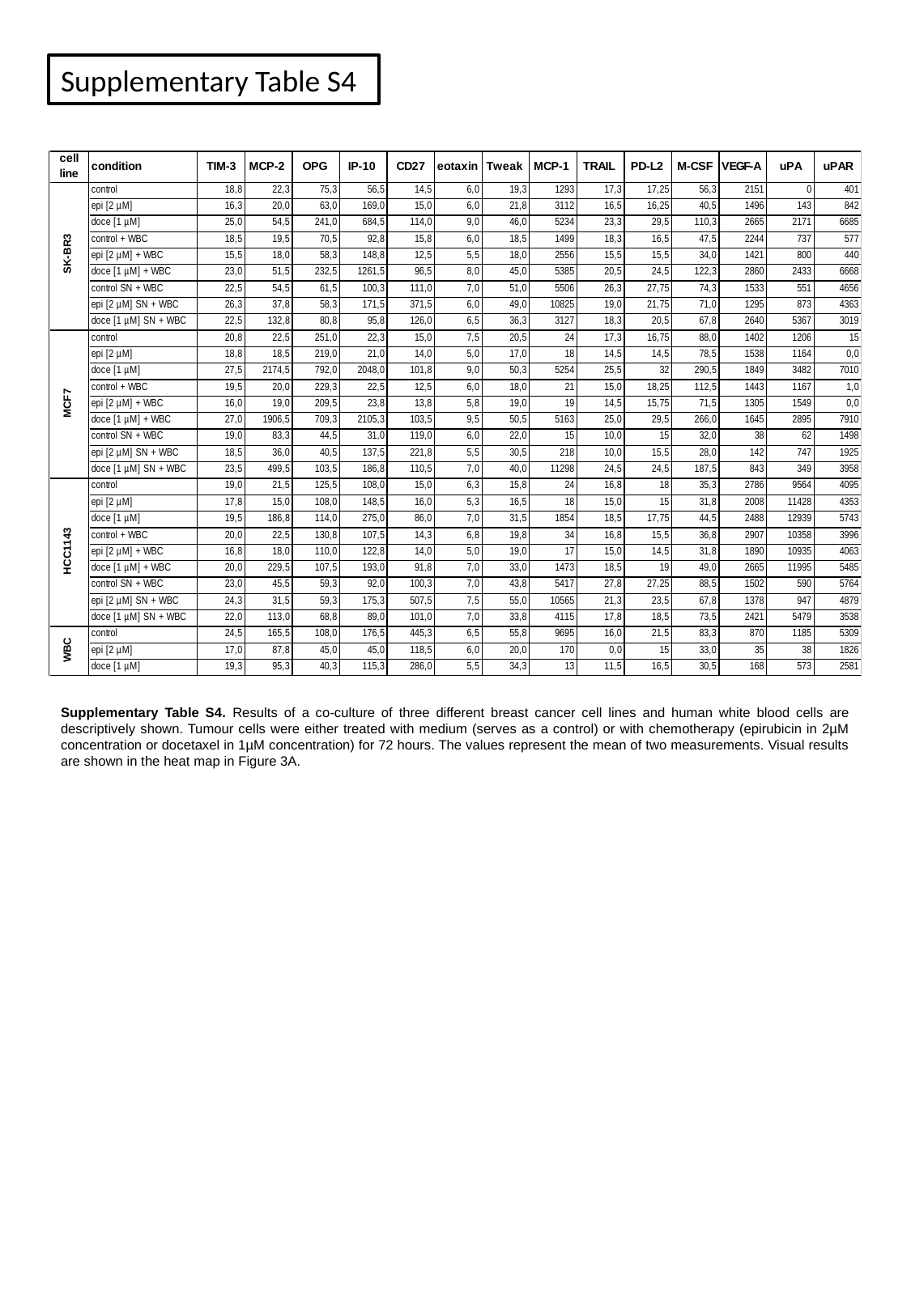

Supplementary Table S4
Supplementary Table S4. Results of a co-culture of three different breast cancer cell lines and human white blood cells are descriptively shown. Tumour cells were either treated with medium (serves as a control) or with chemotherapy (epirubicin in 2µM concentration or docetaxel in 1µM concentration) for 72 hours. The values represent the mean of two measurements. Visual results are shown in the heat map in Figure 3A.

## Slide 5
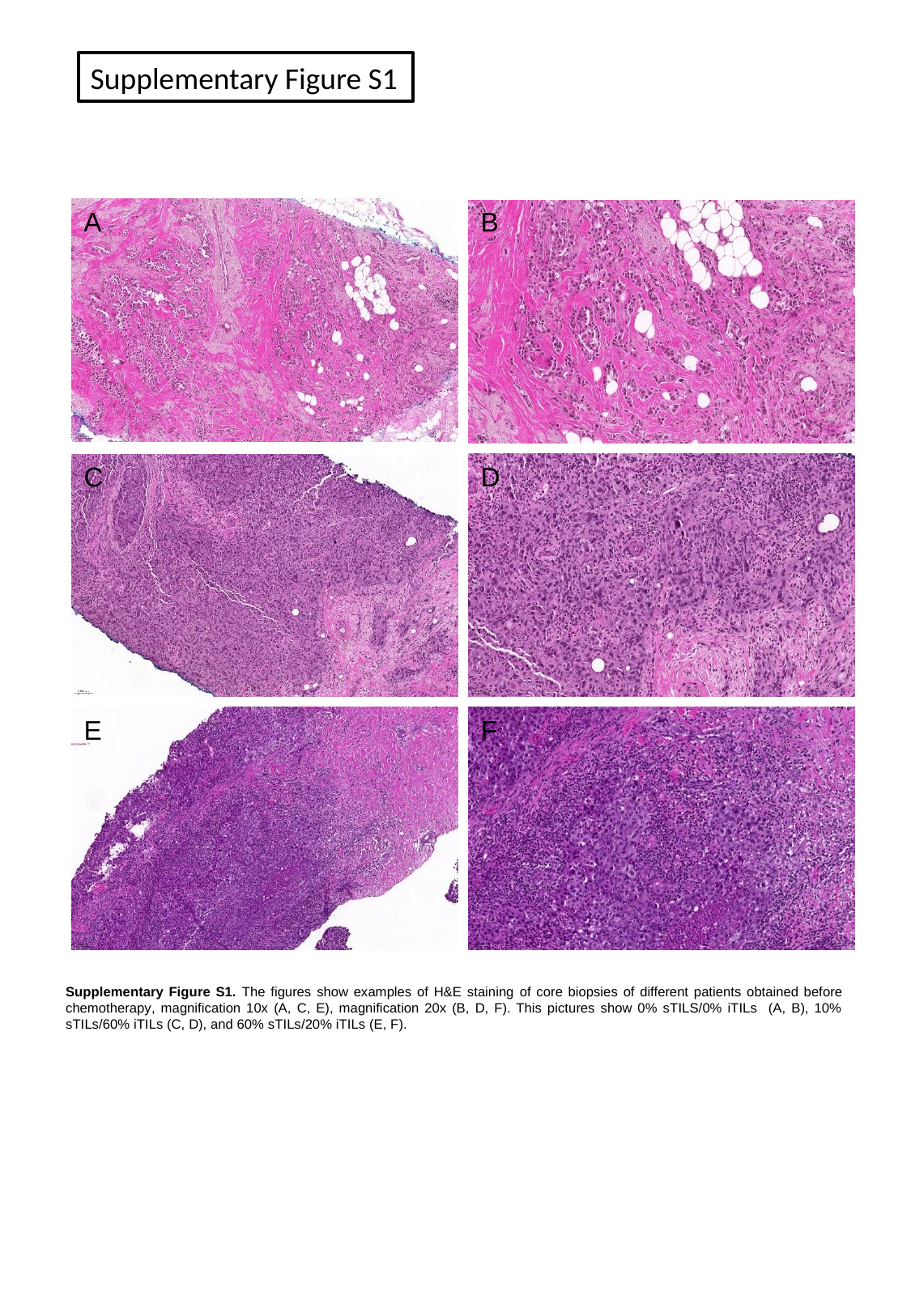

Supplementary Figure S1
A
B
C
D
E
F
Supplementary Figure S1. The figures show examples of H&E staining of core biopsies of different patients obtained before chemotherapy, magnification 10x (A, C, E), magnification 20x (B, D, F). This pictures show 0% sTILS/0% iTILs (A, B), 10% sTILs/60% iTILs (C, D), and 60% sTILs/20% iTILs (E, F).

## Slide 6
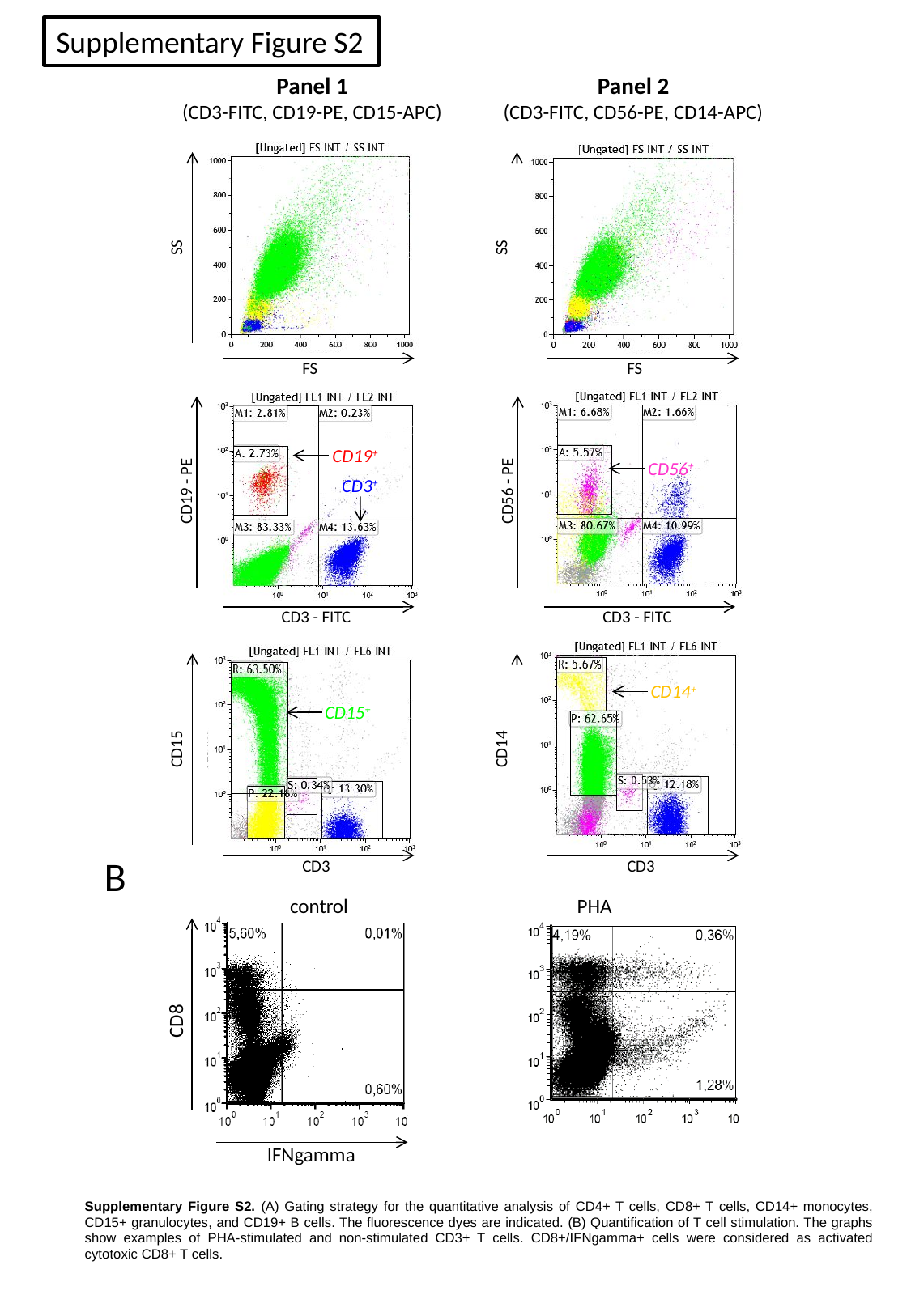

Supplementary Figure S2
Panel 1
(CD3-FITC, CD19-PE, CD15-APC)
Panel 2
(CD3-FITC, CD56-PE, CD14-APC)
SS
SS
FS
FS
CD19+
CD56+
CD3+
CD19 - PE
CD56 - PE
CD3 - FITC
CD3 - FITC
CD14+
CD15+
CD15
CD14
B
CD3
CD3
control
PHA
CD8
IFNgamma
Supplementary Figure S2. (A) Gating strategy for the quantitative analysis of CD4+ T cells, CD8+ T cells, CD14+ monocytes, CD15+ granulocytes, and CD19+ B cells. The fluorescence dyes are indicated. (B) Quantification of T cell stimulation. The graphs show examples of PHA-stimulated and non-stimulated CD3+ T cells. CD8+/IFNgamma+ cells were considered as activated cytotoxic CD8+ T cells.

## Slide 7
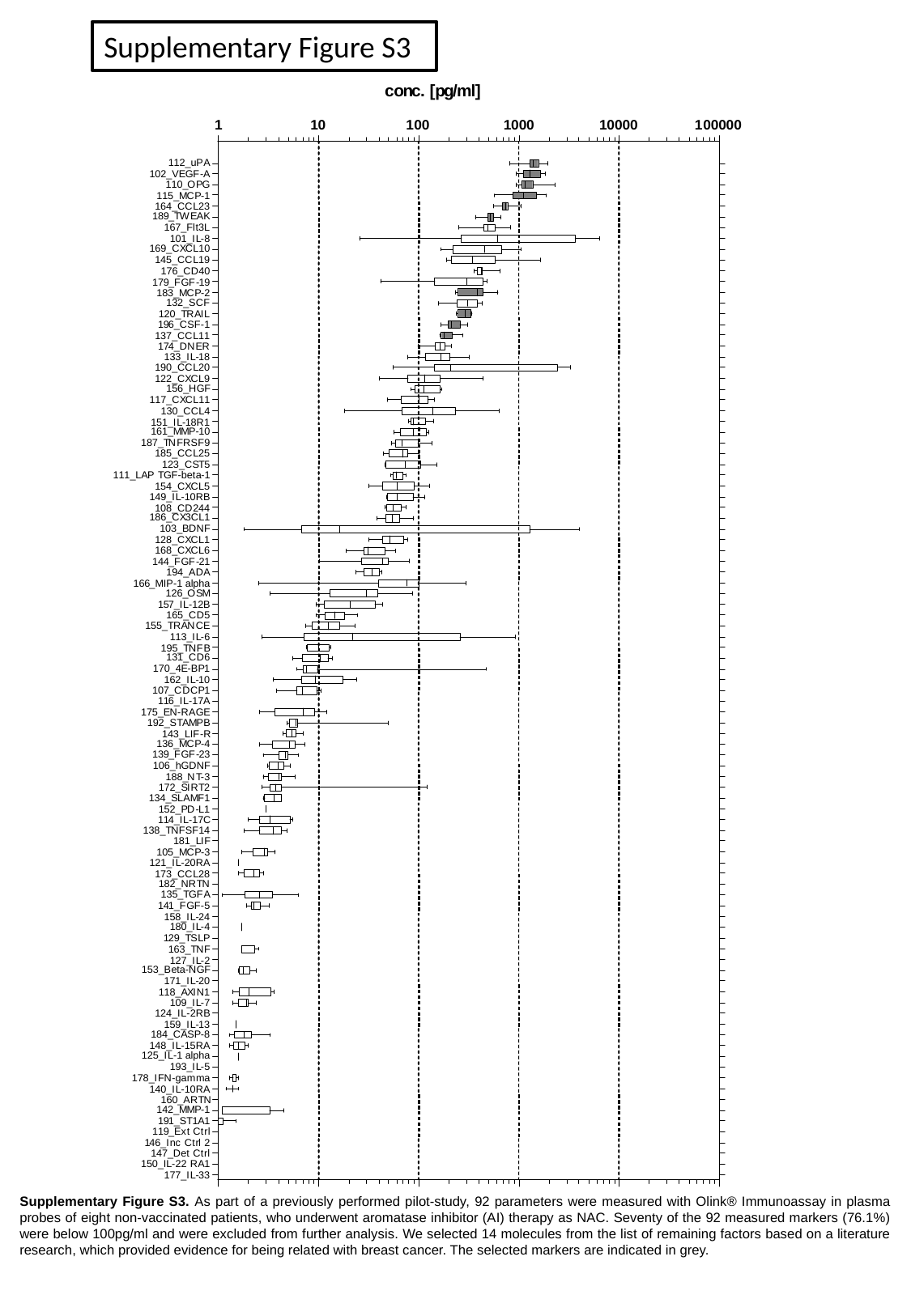

Supplementary Figure S3
Supplementary Figure S3. As part of a previously performed pilot-study, 92 parameters were measured with Olink® Immunoassay in plasma probes of eight non-vaccinated patients, who underwent aromatase inhibitor (AI) therapy as NAC. Seventy of the 92 measured markers (76.1%) were below 100pg/ml and were excluded from further analysis. We selected 14 molecules from the list of remaining factors based on a literature research, which provided evidence for being related with breast cancer. The selected markers are indicated in grey.

## Slide 8
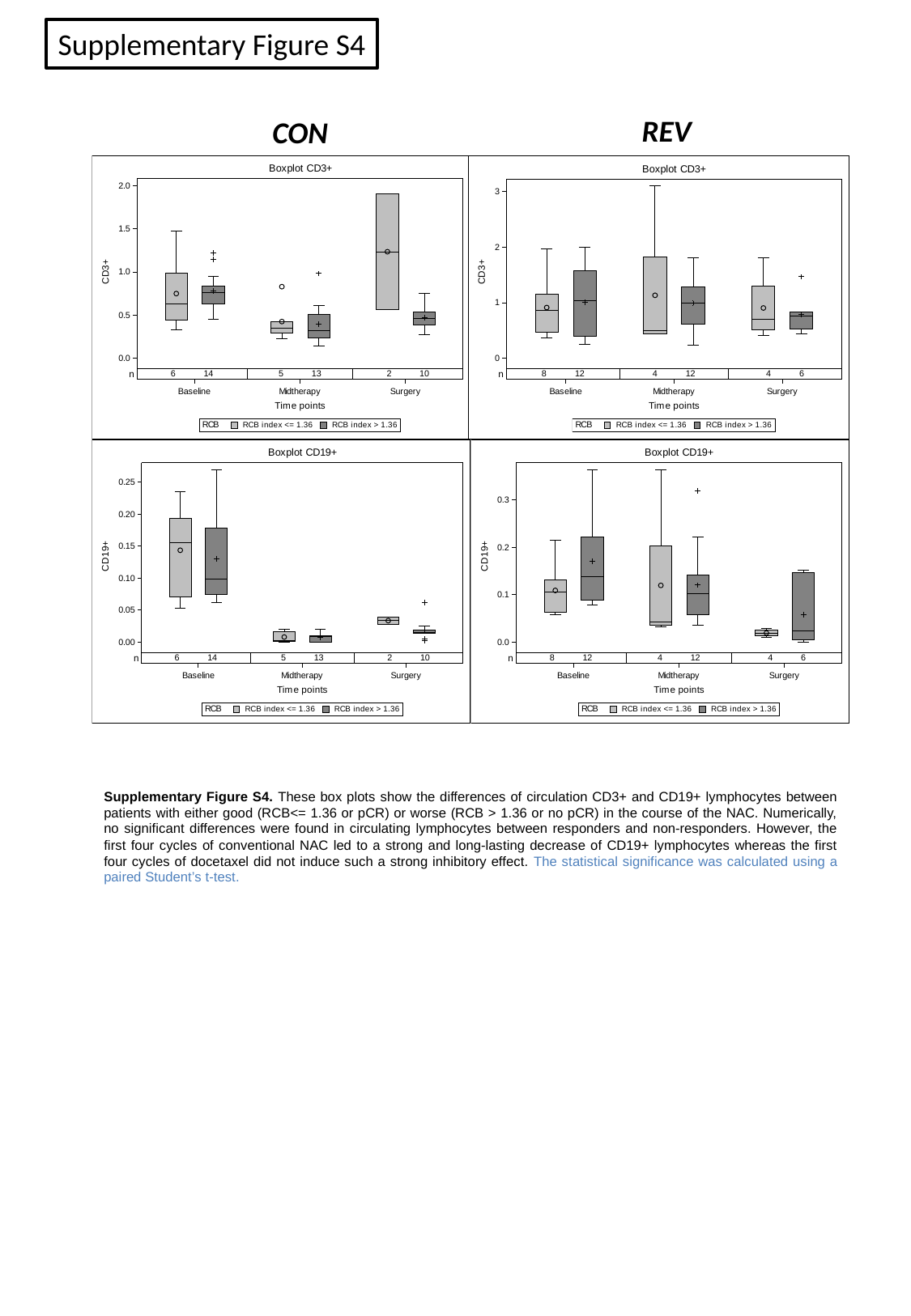

Supplementary Figure S4
REV
CON
Supplementary Figure S4. These box plots show the differences of circulation CD3+ and CD19+ lymphocytes between patients with either good (RCB<= 1.36 or pCR) or worse (RCB > 1.36 or no pCR) in the course of the NAC. Numerically, no significant differences were found in circulating lymphocytes between responders and non-responders. However, the first four cycles of conventional NAC led to a strong and long-lasting decrease of CD19+ lymphocytes whereas the first four cycles of docetaxel did not induce such a strong inhibitory effect. The statistical significance was calculated using a paired Student’s t-test.

## Slide 9
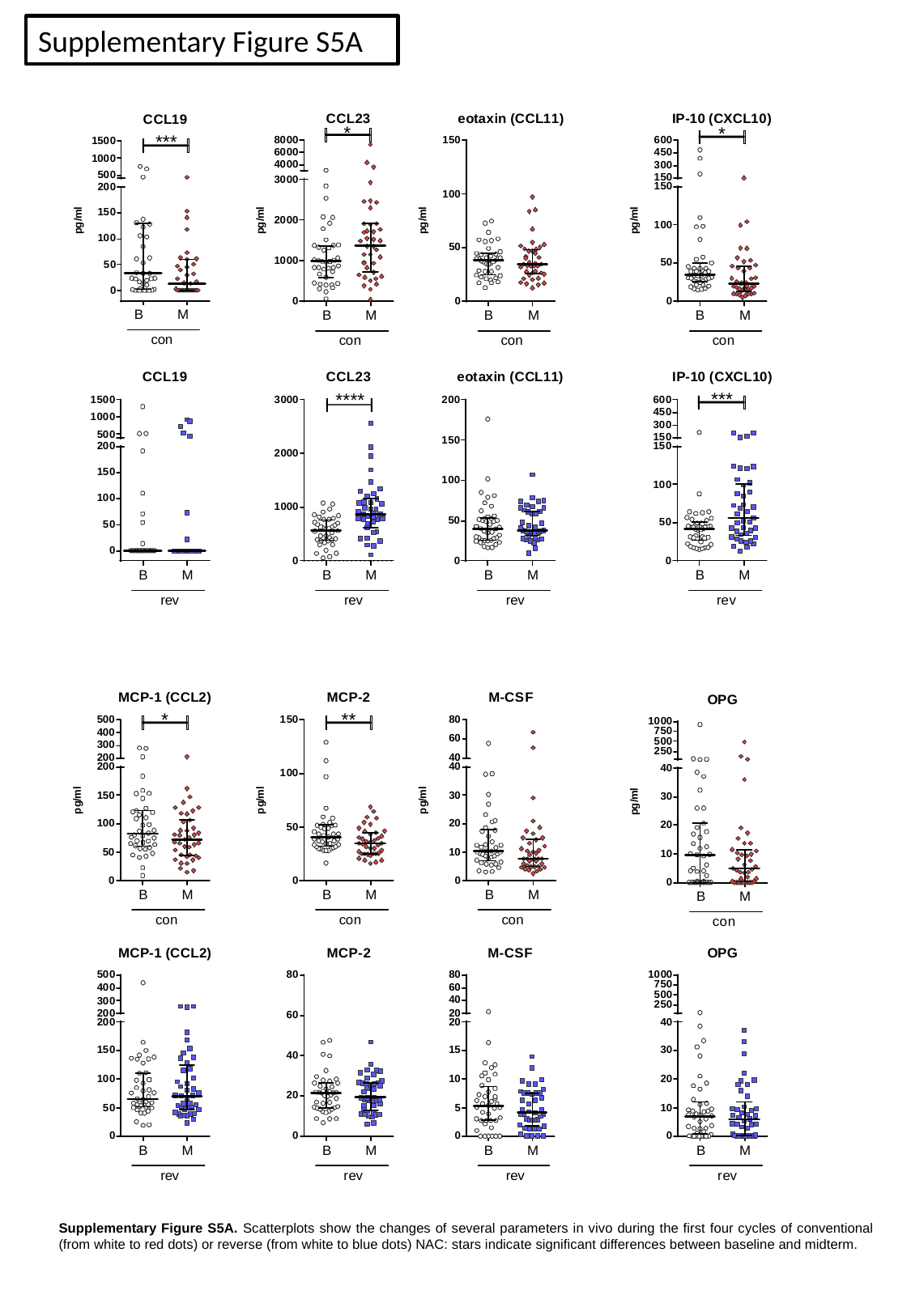

Supplementary Figure S5A
Supplementary Figure S5A. Scatterplots show the changes of several parameters in vivo during the first four cycles of conventional (from white to red dots) or reverse (from white to blue dots) NAC: stars indicate significant differences between baseline and midterm.

## Slide 10
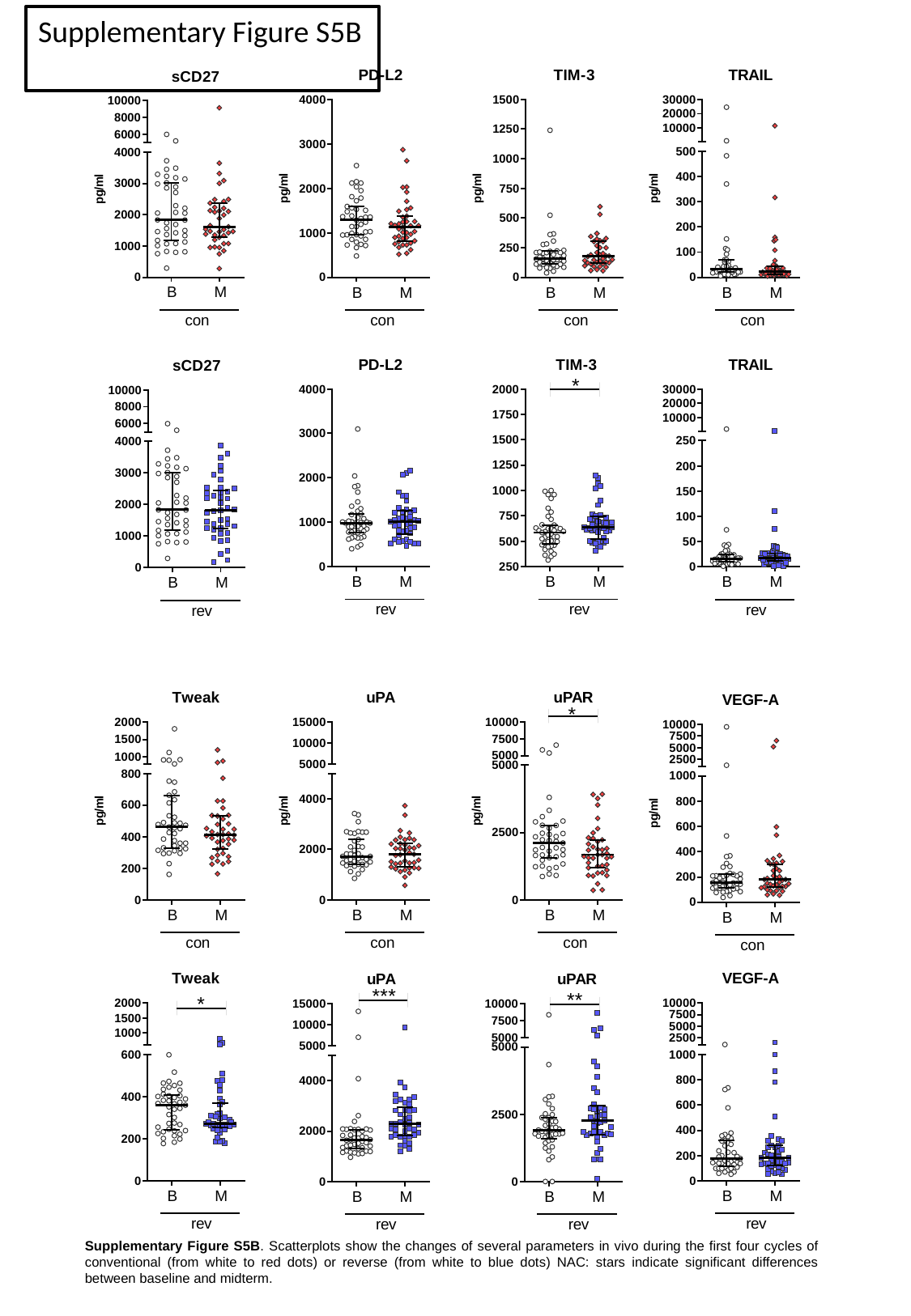

Supplementary Figure S5B
Supplementary Figure S5B. Scatterplots show the changes of several parameters in vivo during the first four cycles of conventional (from white to red dots) or reverse (from white to blue dots) NAC: stars indicate significant differences between baseline and midterm.
